# The Right Partner in Crime: Unlocking the Potential of the Anti-EGFR Antibody Cetuximab *via* Combination With Natural Killer Cell Chartering Immunotherapeutic Strategies

**DOI:** 10.3389/fimmu.2021.737311

**Published:** 2021-09-07

**Authors:** Hasan Baysal, Ines De Pauw, Hannah Zaryouh, Marc Peeters, Jan Baptist Vermorken, Filip Lardon, Jorrit De Waele, An Wouters

**Affiliations:** ^1^Center for Oncological Research (CORE), Integrated Personalized & Precision Oncology Network (IPPON), University of Antwerp, Antwerp, Belgium; ^2^Department of Medical Oncology, Antwerp University Hospital, Edegem, Belgium

**Keywords:** cetuximab, epidermal growth factor receptor (EGFR), natural killer cells (NK cells), combination therapy, immunotherapy, antibody-mediated cellular cytotoxicity (ADCC)

## Abstract

Cetuximab has an established role in the treatment of patients with recurrent/metastatic colorectal cancer and head and neck squamous cell cancer (HNSCC). However, the long-term effectiveness of cetuximab has been limited by the development of acquired resistance, leading to tumor relapse. By contrast, immunotherapies can elicit long-term tumor regression, but the overall response rates are much more limited. In addition to epidermal growth factor (EGFR) inhibition, cetuximab can activate natural killer (NK) cells to induce antibody-dependent cellular cytotoxicity (ADCC). In view of the above, there is an unmet need for the majority of patients that are treated with both monotherapy cetuximab and immunotherapy. Accumulated evidence from (pre-)clinical studies suggests that targeted therapies can have synergistic antitumor effects through combination with immunotherapy. However, further optimizations, aimed towards illuminating the multifaceted interplay, are required to avoid toxicity and to achieve better therapeutic effectiveness. The current review summarizes existing (pre-)clinical evidence to provide a rationale supporting the use of combined cetuximab and immunotherapy approaches in patients with different types of cancer.

## Introduction

The field of cancer treatment has significantly advanced, driven primarily through an increased characterization of the molecular biology, the microenvironment, and the involvement of the immune system in several critical mechanisms of cancer. These advances have led to the development and implementation of targeted and immunotherapies. Targeted therapies are aimed at specifically inhibiting oncogenic signaling pathways that control tumor growth and/or angiogenesis, whereas immunotherapies focus on (re)activating the immune system. Today, both treatment modalities are at the forefront of personalized medicine in cancer treatment.

Several major signaling pathways such as β-catenin, Wnt, phosphatidylinositol 3-kinase (PI3K), and Mitogen-activated protein kinase (MAPK) are recognized for their potentially oncogenic characteristics (1). Among them, the epidermal growth factor receptor (EGFR) is likely the most commonly investigated signaling pathway, renowned for its fundamental role in the tumorigenesis of many cancer types (2). While EGFR expression normally is found between 40 000 to 100 000 receptors/cell (depending on the tissue type), overexpression of EGFR is seen in a majority of cancers, with up to 2 000 000 receptors/cancer cell (3). Thus, downstream signaling of the Ras/Raf/MAPK, PI3K/AKT, JAK/STAT and PLC/PKC pathways is intensified (4), leading to enhanced cellular proliferation, differentiation, survival, migration and motility (5). Inhibition of EGFR has therefore been a compelling topic of research and has led to the development of two classes of anti-EGFR agents: Immunoglobulin G (IgG)-based monoclonal antibodies (mAbs), which competitively bind the ligand-binding site and small-molecule tyrosine kinase inhibitors (TKIs), which compete with adenosine triphosphate (ATP) to bind intracellular EGFR tyrosine kinase domains.

What makes mAbs highly attractive is the ability of IgG1 mAbs to induce antibody-dependent cell-mediated cytotoxicity (ADCC) through Fc receptor-bearing immune cells, increasing tumor immunogenicity and providing a rationale to combine anti-EGFR mAbs with immunotherapies. Cetuximab and necitumumab are the only approved IgG1 mAbs against EGFR (**Table 1**). While cetuximab has been extensively studied in various tumor types (6, 7), literature regarding necitumumab is still limited. Interestingly, similar cytotoxicity has been shown against the DiFi colorectal cancer cell line, due to their affinity for similar EGFR epitopes (8, 9). On the other hand, panitumumab, an IgG2 based anti-EGFR mAb, has similar anti-EGFR activity as cetuximab despite binding different epitopes (10, 11). In monotherapy, the ASPECCT study conducted in chemotherapy-refractory, wild-type *KRAS* metastatic colorectal cancer (mCRC), showed non-inferiority of panitumumab compared to cetuximab (12). Combined treatment of either cetuximab or panitumumab with irinotecan in platinum-refractory mCRC patients similarly suggested non-inferiority (13). Interestingly, studies directly comparing cetuximab and panitumumab in HNSCC have not been conducted. However, while panitumumab failed to improve OS of HNSCC patients in phase II trials in combination with chemoradiotherapy (14, 15) cetuximab, showed clear benefit in both locally advanced and recurrent and metastatic settings and has been granted approval by regulatory authorities herein (16, 17). Therefore, at least in HNSCC, panitumumab, despite having an increased EGFR-affinity, lacks in clinical activity compared to the highly active potential of cetuximab. A possible reason for this may be explained by the differences linked to the IgG backbone.

**Table 1:** Summary of approved EGFR-targeted mAbs.

Drug (Trade name)	Company	Indication	Approval FDA/EMA	Isotype	Recommended dose	Clinical trials*
Cetuximab (Erbitux)	Bristol-Myers Squibb	HNSCC,CRC	2004	Chimeric IgG1	I.V. 400 mg/m^2^ initial, 250 mg/m^2^ weekly	NCT00004227
						NCT00122460
Panitumumab (Vectibix)	Amgen	CRC	2006/2007	Human IgG2	I.V. 6 mg/kg biweekly	NCT00364013
						NCT00115765
Necitumumab	Eli Lilly and	NSCLC	2015/2016	Human	I.V. 800 mg twice in a	NCT00981058
(Portrazza)	Company			IgG1	3-week cycle	NCT01769391

CRC, colorectal cancer; EGFR, epidermal growth factor receptor; EMA, European Medicines Agency; FDA, Food and Drug Administration; HNSCC, head and neck squamous cell carcinoma; I.V., intravenously; NSCLC, non-small cell lung cancer. *Clinical trials upon which approval was based.

As evidenced by prior research, chemotherapeutic agents have immunomodulatory effects, causing (in)direct activation of immune cells due to the release of tumor antigens and certain “danger” signals (18, 19). Targeted therapies are similarly able to reshape the tumor immune microenvironment (TIME) and stimulate the induction of an immune response (20). Immunosurveillance, i.e. the recognition and elimination of malignant cells by the immune system (21), is crucial towards cancer prevention and evasion of immunosurveillance is one of the cancer hallmarks. As the immune system is a complex network of humoral and cellular interactions, alterations in many components of the innate and adaptive immunity lie at hand for tumor evasion (22). In addition, selective survival of tumor cells with a decreased immunogenicity contributes to an evasive tumor growth (23). In this regard, the TIME of several cancers has been characterized, showing both dysfunctional immune cells and a suppressive environment as the main reason for an impaired antitumor immunity (24–27). Based on these principles, immunotherapy has now become a major focus of research in oncology and has led to the implementation of immune checkpoint inhibitors, which have the potential to reawaken silenced immune responses. Recently, several immune checkpoint inhibitors have demonstrated durable response rates and gained Food and Drug Administration (FDA) and European Medicines Agency (EMA) approval for use in several oncological indications, including metastatic melanoma, non-small cell lung carcinoma (NSCLC), renal cell carcinoma, head and neck cancer (HNSCC) and colorectal cancer (CRC) (28–30). In the context of EGFR, besides its oncogenic role, EGFR is involved in three main immune-related processes. These include: (1) repression of antigen presentation *via* downregulation of major histocompatibility complex (MHC) class I and II expression (31); (2) programmed cell death protein (ligand) 1 (PD-1/PD-L1) pathway activation (32); and (3) secretion of immunosuppressive cytokines, such as vascular endothelial growth factor (VEGF), and interleukins (IL) IL-6 and IL-10 (33, 34). Therefore, the use of anti-EGFR therapeutics, such as cetuximab, is a promising strategy of altering the TIME towards tumor recognition and potentially killing rather than evasion and tumor growth.

Although both targeted and immunotherapies are successfully implemented into clinical practice, they present some limitations. In general, when immunotherapies are successful, they can achieve long-term responses in patients. However, response rates with immunotherapies are typically low. In contrast, targeted therapies can achieve much higher initial responses but are lacking in long-term tumor remission, due to the development of resistance. Therefore, growing evidence suggests that combining targeted therapies with immunotherapies can achieve much greater clinical effectiveness for a larger patient population. However, since tumor types vary greatly in their TIME, the applicability of these combinations is dependent on the tumor type and severity of disease (35, 36). For instance, under healthy conditions, all nucleated cells will express MHC class I “self” antigens as a measure of host and non-threat recognition. However, tumor cells often will decrease the expression of MHC-I to evade T-cell recognition of tumor antigens and also their effector functions (37). Therefore, the applicability of T cell-focused immunotherapies is currently complicated by the inability of T cells to recognize MHC-I^neg^ tumors as well the requirement of neoantigens for the induction of adequate responses. These shortcomings may potentially be circumvented by the innate counterpart of T cells, the natural killer (NK) cells, as they can recognize tumor cells independent of their MHC status and require no presentation of neoantigens. Moreover, NK cell responses can further shape the TIME towards activation of the adaptive immunity, and thus are key effectors of antitumor immunity. In addition, although NK cell infiltration is not equal in all tumor types, the number of tumor-infiltrating NK cells (TINK) has been associated with a significantly better outcome in many tumor types (29, 38–40). Monteverde et al. and others showed that in addition to the number of NK cells, the level of *ex vivo* antibody-dependent cell-mediated cytotoxicity (ADCC) induction can be used as a predictive biomarker for cetuximab treatment in the clinic (41–43). Together, this shows a unique opportunity for NK cell-based immunotherapy together with anti-EGFR targeted therapeutic approaches to re-establish functional NK cell responses, prime the TIME for the adaptive immunity, and generate more durable antitumor responses.

In this review, we will briefly describe the fundamentals of NK cell biology and functionality followed by a comprehensive review of combination strategies involving EGFR targeted therapies together with immunotherapeutic modalities that aim to restore/enhance the antitumor effects of NK cells. We will focus on cetuximab as an anti-EGFR targeted mAb, as its immune activity has been studied extensively both in monotherapy as well as in combination with other molecules. However, the efficacy of anticancer drugs varies significantly among different tumor types. Therefore, similar or possibly improved results could be achieved with other mAb-based immunotherapies following careful examination and characterization of the TIME.

## NK Cell Biology and Antitumor Activity

Grouped among the population of lymphocytes, NK cells share the same progenitor as T and B lymphocytes but differentiate themselves through an antigen-independent activation (44). While the effector function of NK cells overlaps with CD8^+^ T cells, they do respond to different stimuli and thus complement each other in settings where the effectiveness of one is lacking. Therefore, NK cells, as part of the innate immune system, form the first line of defense against cancer and pathogens (45). In humans, NK cells make up roughly 10-15% of all immune cells (46) and are defined as CD3^-^ CD56^+^ (47). The two major NK subpopulations are termed CD56^bright^ (high cytokine producers) and CD56^dim^ (high cytotoxicity) NK cells. About 90% of circulating and splenic NK cells are CD56^dim^, while CD56^bright^ NK cells are mostly present in the secondary lymphoid organs (48). Notably, CD56^bright^ NK cells make up the largest portion of tumor-associated NK cells in several tumor types (48, 49).

**Figure 1:**
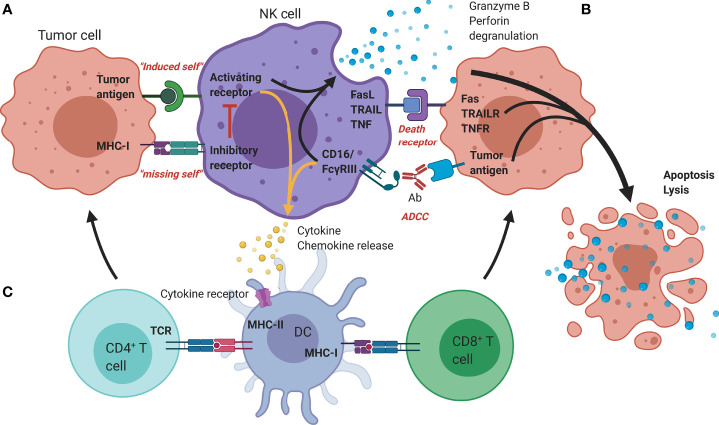
Mechanisms of antitumor functionality of NK cells. **(A)** The represented ‘activating’ and ‘inhibitory’ NK cell receptors determine the NK cell activation through interaction with; (i) stress-induced tumor antigens or ligands for activating receptors acting towards an ‘induced-self’ response or (ii) MHC-I self-antigens or ligands for inhibitory receptors. **(B)** Additional tumor killing can be induced through either death receptors (FAS/TRAILR/TNFR), or antibody-dependent cellular cytotoxicity (Granzyme B/perforin degranulation). **(C)** Additional immune modulation by NK cells occurs through secretion of cyto-/chemokines that promote DC maturation and allow crosstalk with T cells, facilitating the induction of an adaptive immune response. Ab, Antibody; DC, Dendritic cell; FasL, Fas ligand; MHC, Major histocompatibility complex; NK, Natural killer; TCR, T-cell receptor; TNF(R), Tumor necrosis factor (receptor); TRAIL(R), TNF-related apoptosis-inducing ligand (receptor).

**Figure 2:**
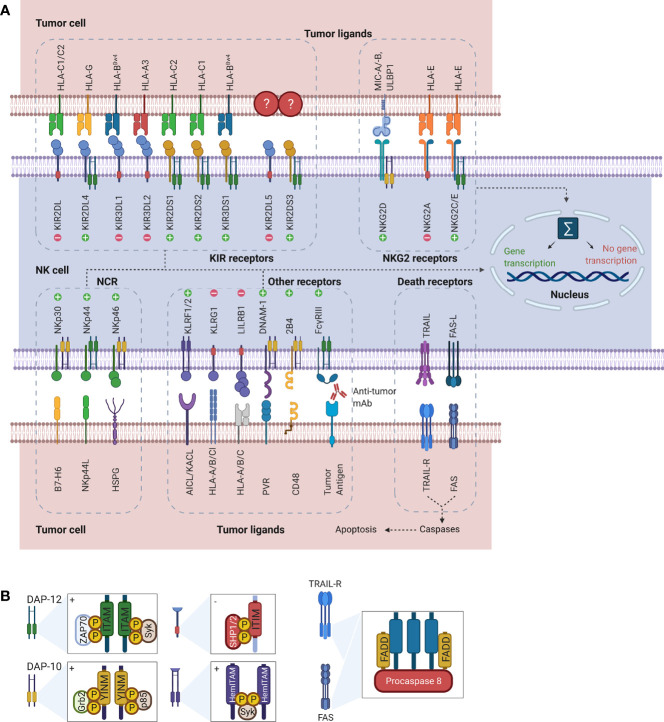
Representation of NK cell receptor-ligand interactions and signaling motifs that enable downstream cell signaling. **(A)** The most common NK cell receptor families are illustrated together with their ligands. While some receptors engage multiple ligands, others such as KIR2DL5 and KIR2DS3/S4/S5 have no known ligands. Interaction of ligands with receptors causes activation of downstream signaling pathways. Depending on the type of receptor, this may cause either activation of gene transcription or suppression. **(B)** Downstream signaling is activated through processing of the receptors-ligand interaction through signaling motifs. Symbols “+” and “-” in the boxes indicate activating and inhibiting signaling. While ITAM and YINM signaling motifs are bound to DAP-10 and -12 adaptor protein respectively, ITIM and HemITAM are present on the receptors and do not require adaptor proteins. The death receptors Fas and TRAIL-R signal through FADD to induce induction of apoptosis in tumor cells. Downstream signaling and gene transcription leading to NK cell activation is dependent on the sum of all activating and inhibiting signals. AICL, Activation-induced C-type lectin; DAP, DNAX-activating protein; DNAM, DNAX accessory molecule; FADD, Fas-associated protein with DD; Grb2, Growth factor receptor-bound protein 2; HemITAM, Hemi-immunoreceptor tyrosine-based activation motif; HLA, Human leukocyte antigen; HSPG, Heparan sulfate proteoglycans; ITAM, Immunoreceptor tyrosine-based activation motif; ITIM, Immunoreceptor tyrosine-based inhibitory motif; KACL; Keratinocyte-associated C-type lectin, KIR, Killer cell immunoglobulin-like receptor; KLRF/G, Killer cell lectin-like receptor F/G; LILRB1, Leukocyte immunoglobulin-like receptor B1 MICA/B, MHC class I polypeptide–related sequence A/B; NCR; Natural cytotoxicity receptors; NK, Natural killer; NKG2, Natural killer group 2; PVR, Poliovirus receptor; SHP1/2, Src homology region 2 domain-containing phosphatase-1; Syk, Spleen tyrosine kinase; TRAIL(R), TNF-related apoptosis-inducing ligand (receptor); ULBP, UL16 binding protein; YINM, Tyrosine-based signaling motif; ZAP70, Zeta-chain associated protein kinase.

Rather than depending on prior antigen presentation, NK cell immunosurveillance is based on a balance between interaction of activating and inhibitory receptors on their surface (50). In this regard, ‘the missing self’ principle (51) describes activation of NK cells through a decreased expression of MHC class I on tumor cells. However, lack of self-recognition alone does not determine NK cell activation and therefore the ‘induced self’ hypothesis describes the requirement of tumor antigens or ligands of activating receptors to be expressed in addition to a reduced self-recognition to establish NK cell activation (**Figure 1A**) (52, 53). A prerequisite for NK cell cytotoxicity is the formation of an immunological synapse, a tight and complex junction formed between an NK cell and its target cell (54). Importantly, FcγRs range in their affinity for human IgGs. The high-affinity FcγRI are therefore able to bind monomeric IgGs while other FcγRs have a low-affinity and are only able to interact with multimeric IgG complexes (55, 56). Following interaction with activating signals, numerous cellular molecules (including receptors, signaling molecules and cellular organelles) will induce cytoskeletal reorganization of NK cells and polarize lytic granules, filled with pore-forming proteins (perforin) and serine proteases (granzymes), towards the synaptic site. Targeted exocytosis of these granules into the synaptic space induces apoptosis in the target cell (57). NK cell activation may occur following interaction with death receptors such as first apoptosis signal (Fas) receptor and tumor necrosis factor (TNF)- related apoptosis-inducing ligand receptor (TRAIL-R) with their ligands, FasL and TRAIL, respectively (**Figure 1B**) (58, 59). In addition, various groups of inhibitory and activating NK cell receptors exist as well, as shown in **Figure 2A**. Inhibitory NK cell receptors that can recognize MHC-I antigens on tumor cells include the killer Ig-like receptors (KIR2DL and KIR3DL), C-type lectins NK cell group 2 (NKG2A/B) subfamily and leukocyte immunoglobulin-like receptors (LILR) (60, 61). In addition, immune checkpoint receptors, such as cytotoxic T-lymphocyte-associated protein 4 (CTLA-4), PD-1, and the T cell immunoreceptor with Ig and ITIM domains (TIGIT) are present on NK cells as well and prevent sustained activation through inhibitory signaling (62, 63). Interestingly, several of the activating cell surface receptors on NK cells are derived from the same receptor families as their inhibitory counterparts. For example, KIR2DS and KIR3DS belong to the KIR family receptors, while NKG2C/D belong to the C-type lectin family (64). Additionally, the family of natural cytotoxicity receptors (NCR), i.e. NKp46, NKp30, and NKp44, can recognize a broad spectrum of ligands ranging from viral-, parasite- and bacterial-derived to cellular ligands (65). Downstream signaling of NK cell receptors is dependent on the interaction between activating and inhibiting signaling motifs. Activating receptors associated with DNAX-activating protein 10 or 12 (DAP-10/-12) process signals through tyrosine-based signaling motif (YINM) or tyrosine-based activation motif (ITAM) respectively (66). Meanwhile, Inhibitory receptors carry the immunoreceptor tyrosine-based inhibitory motif (ITIM) that overrides DAP-10/-12 signaling and consequently prevents NK cell activation (**Figure 2B**) (67).

**Figure 3:**
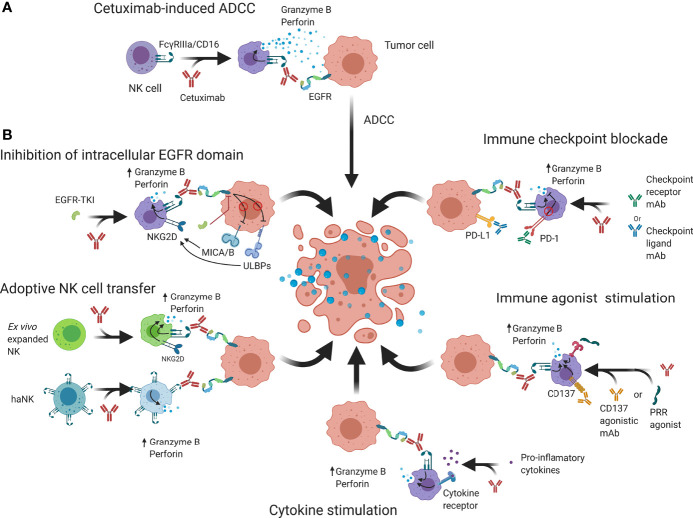
Schematic overview of possible strategies that may be employed to enhance cetuximab-based anticancer NK cell responses. **(A)** Cetuximab is a mAb that interacts with FcγRIIIa/CD16 receptors on NK cells and EGFR on tumor cells to abrogate EGFR signaling and induce granzyme B and perforin release, causing cell death. **(B)** NK cell cytotoxicity may be enhanced by additional binding of intracellular EGFR kinase domains that can regulate expression of NK cell receptors. Genetically engineered NK cells such as haNK or CAR-NK have increased natural cytotoxicity and activating signaling which through adoptive transfer can enhance ADCC. Immune checkpoint blockers prevent suppression of NK cell functions by reducing inhibitory signaling while immune agonists aim to increase activating signals. Cytokine stimulation increases NK cell functions and allows an enhanced ADCC response to take place. ADCC; Antibody-dependent cell-mediated cytotoxicity; CAR, Chimeric antigen receptor; EGFR, Epidermal growth factor receptor; IL-2/12/15/21; Interleukin 2/12/15/21; MICA/B, MHC class I polypeptide–related sequence A/B; NKG2D, Natural killer group 2D; PD-1, Programmed cell death protein 1; PD-L1, Programmed death-ligand 1; PRR; Pathogen recognition receptors; ScFv, Single-chain variable fragment TKI; Tyrosine kinase inhibitor; ULBP, UL16 binding protein.

Besides direct receptor-ligand interaction, NK cells can become activated by interaction of their Fc receptors (FcγRIIIa/CD16) with the Fc-domain of immunoglobulin G (IgG) antibodies. To achieve subsequent tumor cell killing, the antibody Fab-domain must bind its target on tumor cells to initiate NK cell cytokine and cytotoxic granule secretion, thus inducing ADCC (**Figures 1B** and **Figure 3A**) (68). Interestingly, ADCC dysfunction has been linked to cancer progression and forms an important mechanism of action for therapeutic mAbs (68, 69). Among the IgG subtypes that have been identified and used to generate antitumor therapies, IgG1-based mAbs have the highest potency to bind with CD16 and thus induce the highest ADCC responses (70). This is evident when comparing clinical data of cetuximab (IgG1) with panitumumab (IgG2) indicating that, although both effectively inhibit EGFR signaling, cetuximab mediates a greater extent of immune-related activity (10, 71). Preclinical models in CD16 deficient mice observed similar antitumor responses between cetuximab and panitumumab due to inhibition of EGFR (72, 73). However, CD16 wild-type mice consistently had enhanced antitumor responses with cetuximab which were linked to its IgG1 backbone (73, 74).

Aside from IgG subtypes, other factors related to interindividual heterogeneity rather than the composition of the mAb can affect ADCC by NK cells. First, FcyRIIIa gene polymorphisms are the most well-known factor in this regard. Individuals possessing the 158V/V allotype induce higher ADCC responses in various tumor types compared to individuals with the 158V/F or 158F/F allotypes (42, 75, 76). **In vitro**, transduction of the human NK-92 cell line with the 158V/V allotype (high-affinity NK; haNK) increased natural cytotoxicity, cetuximab-induced ADCC (77) and cytokine secretion (78). Second, the presence of immunosuppressive cytokines, such as transforming growth factor β (TGF-β) or IL-10 or increased signaling through inhibitory KIR receptors or NKG2A, provides additional inhibitory signals. This shifts the balance of NK cell activity towards an inhibitory state, preventing the induction of ADCC (79–81). Third, while cetuximab resistance mechanisms limit the effectiveness of anti-EGFR treatments and promote tumor cell survival, they are unable to prevent granzyme B (GZMB)-induced apoptosis by healthy NK cells following cetuximab treatment (82–85). On the other hand, EGFR-independent resistance mechanisms against immune cell-mediated cell death have been described (86). For example, the presence of tumor cells expressing serine protease inhibitor-9 (PI-9), an irreversible inhibitor of GZMB, correlated with a poorer outcome in melanoma patients (87, 88). Overexpression of X-linked inhibitor of apoptosis protein (XIAP), a potent caspase inhibitor, in breast cancer induced resistance to cetuximab-mediated ADCC in both a caspase-dependent and -independent manner (*via* accumulation of reactive oxygen species) (89). Lastly, activation of autophagy under hypoxic conditions showed beclin-1-mediated degradation of NK cell-derived GZMB *in vitro*, which compromised the ability of NK cells to eliminate breast cancer cell lines (90). Notwithstanding these variable factors, the ability for ADCC remains a valuable and promising option in the therapeutic armamentarium, favoring mAbs such as cetuximab.

Next to direct activation, indirect NK cell activation primes NK cells towards activation by increasing the expression of activating receptors, reducing the threshold for activation and reducing the responsiveness to inhibitory signals (91). This can be achieved through interaction with mature dendritic cells (DC) or cytokines such as IL-2, IL-12, IL-15, IL-18, IL-21 and type-I interferons (92, 93). However, vice versa, activated NK cells can cross-talk with DC, promoting their maturation and subsequent CD8^+^ T cell priming, resulting in the generation of tumor-specific T cells that contribute to the antitumor immune reaction (**Figure 1C**) (94). As such, in addition to tumor elimination, NK cells also modulate and shape antitumor immunity, showing their crucial role to achieve tumor elimination.

## Strategies to Enhance Cetuximab Driven Immune Activity

Initial preclinical models showed that efficacy of cetuximab on inhibition of the downstream effectors and interfering with tumor cell proliferation could be further enhanced through combination with conventional therapies, such as radiotherapy or chemotherapy (16, 95, 96). Later, it became apparent that this enhancement was in part attributable to an immunological response through an enhanced tumor infiltration of immune cells and ADCC (97, 98). However, there is evidence to suggest that TINK cells reside in an impaired state and only induce limited activity (99, 100). Furthermore, NK cell immune evasion by tumor cells has been described to be caused through two main mechanisms: (1) reduction of activating ligands on tumor cells; and (2) a dominance of NK cell inhibitory signals, preventing downstream signaling of activating signals. In addition, additional immunosuppressive mechanisms from bystander regulatory immune cells can further stimulate tumor progression (101). Therefore, in describing NK cell immunosurveillance enhancements, applicability depends on the composition of the TIME of different tumor types. Re-establishing NK cell functionality thus is a topic of great interest, as it could improve the antitumor immune responses observed in the clinic. In this regard, the research mainly focuses on two major approaches: (1) increasing signaling through immunoreceptor tyrosine-based activation motif (ITAM/YINM)-containing receptors; and (2) decreasing signaling and cross-linking of inhibitory motif (ITIM)-containing receptors. Below, we discuss several strategies to potentiate NK cells to elevate cetuximab efficacy to the next level (**Figure 3B**).

### Dual Inhibition of EGFR Extracellular and Intracellular Domains

Despite initial promising results observed with anti-EGFR treatments, the most prominent limiting factor of its clinical effectiveness is the presence/development of drug resistance. Research has considerably focused on unraveling mechanisms behind this resistance and results have shown various ways to prevent/overcome EGFR-resistance (102–105). Of these, simultaneous inhibition of extracellular and intracellular domains of EGFR has been suggested to increase the overall antitumor effects. In this regard, the combined use of cetuximab and erlotinib/gefitinib induced synergistic antitumor effects with decreased proliferation and increased apoptosis in various human cell lines (106, 107). Phase I/II trials in NSCLC and CRC using combined treatment of cetuximab with gefitinib or erlotinib reported no additional toxicities with moderately enhanced antitumor effects (107–110). Even better results were obtained with second (afatinib) and third-generation (osimertinib) anti-EGFR TKIs in combination with cetuximab (111, 112). Additionally, the sequential treatment of NSCLC patients in a phase I trial using sequential treatment of afatinib and cetuximab observed improved objective response rates and progression-free survival (PFS) (111).

Besides an improved antitumor effect, it was also suggested that combined targeting of extracellular and intracellular domains of EGFR could improve immunologic responses. While the immunological effects of mAbs are well described, the therapeutic effect of EGFR TKIs has been predominantly attributed to the inhibition of signal transduction. However, current knowledge suggests that TKIs might indirectly be involved in antitumor immune responses. For example, treatment of NSCLC and CRC cells with the anti-EGFR TKI gefitinib or erlotinib increased natural cytotoxicity of NK cells through upregulation of NKG2D ligands ULBP-1/-2 and MHC class I polypeptide–related sequence (MIC)A/B (113–116), and downregulation of PD-L1 expression (117). In contrast, another study reported downregulated MICB and ULBP–2/5/6 expression following treatment with erlotinib (118). This indirect immunomodulatory effect suggests that simultaneous inhibition of extracellular and intracellular EGFR domains could increase antitumoral effects, due to dual targeting of EGFR, and improve immunologic responses as well. Indeed, combined treatment with cetuximab and erlotinib improved NK cell activity in NSCLC cell lines and an NSCLC mouse model through an improved ADCC response (119). This is likely caused by an increased expression of the NKG2D ligands by EGFR-TKI (119), which shifts the balance towards NK cell activity. Together with cetuximab-induced ADCC, this shift increases the overall cytotoxic activity of NK cells. A similar study in ovarian cancer cell lines observed enhanced antitumor responses and increased sensitivity towards cetuximab-induced ADCC following treatment with either erlotinib or gefitinib, even in tumor cells that were either intrinsically or acquired resistant to either TKI treatment (120).

One key consideration is the potential for overlapping toxicities of dual EGFR inhibition. However, most trials observe manageable toxicities, with one trial in particular reporting a similar percentage of grade 3/4 adverse events (AEs) when afatinib was combined with cetuximab simultaneously compared to sequential treatment or either treatment alone. However, the overall incidence of AEs was higher in the combination regimen (121). As clinical doses are based on toxicity and not target inhibition, the tolerable doses of each agent in the combination may be suboptimal. However, further clinical investigation is warranted to compare the observed toxicity profile with the effectiveness of this combination.

### Adoptive Transfer Therapy Using (un)Modified NK Cells

#### Adoptive Transfer of Autologous Expanded NK Cells

As NK cells are often impaired in cancer patients, the use of adoptive NK cellular immunotherapy aims to restore NK cell functionality through supplementation or complete replacement of the NK cell populations with functionally active NK cells. As a result, tumor load, and the immunosuppressive TIME could be reduced. Earliest attempts of adoptive NK cell transfer failed to show meaningful clinical responses using *ex vivo* purified and unstimulated NK cells (122).

Therefore, combination of an NK cellular product with cetuximab could enhance the functionality of these NK cells and achieve overall responses through the induction of ADCC. A phase I trial in CRC administered *ex vivo* expanded patient-derived NK cells following cetuximab treatment (123). Noteworthy, the majority of expanded NK cells showed high expression of NKG2D and CD16, and high lymphocyte-activation gene 3 (LAG-3) and TIGIT expression. Cytotoxic effects toward the tumor remained elevated up to 4 weeks following NK cell administration, indicating a favor towards NK activation rather than inhibition. Addition of expanded NK cells following cetuximab treatment displayed an increased cytotoxic activity against tumor cell lines and reduced overall tumor size of heavily pretreated cetuximab-resistant patients. Lastly, patients treated with expanded NK cells following cetuximab showed enriched levels of circulating interferon gamma (IFNγ) and reduced Treg frequencies, suggesting an induction of a Th1‐type adaptive immune response (123).

#### Adoptive Transfer of Allogeneic Expanded NK Cells

With the increased understanding of self-regulation in NK cells, a possible alternative for the limited number of patient-derived NK cells has been the use of allogeneic NK cells. This approach may hold several benefits including the ability to obtain NK cells from healthy donors which may retain greater antitumor activity and the development of off-the-shelf application due to easier and greater availability of NK cells (124). Furthermore, several models to predict alloreactivity of NK cells (graft-*versus*-host disease) have been described (125), the ‘Receptor–ligand mismatch’ model remains one the most established predictive models. Briefly, donor NK cells bearing inhibitory KIR for which the corresponding HLA ligands are missing in the recipient become uninhibited. The presence of (non-HLA-restricted) activating signals can then induce alloreactivity (126, 127).

Sources for alloreactive NK cells include (i) acquiring umbilical cord blood (128),; (ii) partially KIR/HLA matched peripheral blood (126); or (iii) engineered NK cell lines (129). Investigations using the former primary NK cells yielded increased expression of activation markers CD69 and CD16 and strong ADCC responses towards NSCLC and B cell lymphoma *in vitro* and *in mice* (128). Adoptive transfer of the modified NK-92 cell line (haNK) cells with cetuximab harbored the capacity to efficiently kill HNSCC tumor cells in a dose-dependent manner and enhanced ADCC response (130, 131). In a clinical trial in NSCLC, *ex vivo* stimulated KIR/HLA matched healthy donor NK cells were administered together with cetuximab. This combination led to a significantly improved PFS and OS compared to cetuximab alone (132). A phase I trial in gastrointestinal carcinoma used allogeneic IL-2 stimulated NK cells in combination with cetuximab and obtained beneficial clinical responses and a tolerable safety profile (133). Interestingly, while addition of adoptive NK cells increased the number of circulating lymphocytes (CD8^+^, CD4^+^, B and NK cells), cetuximab alone, albeit to a lesser degree, was also able to significantly increase lymphocyte levels. This suggests that part of the increased levels may be related to improvement of cellular immunity and prevention of apoptosis of T cells. Indeed, levels of IFNγ and pro-inflammatory cytokines were significantly more present through combination of cetuximab with adoptive NK cell transfer, indicating an enhanced Th1-response (132). These first and promising results of cetuximab stimulating adoptive NK cell therapy in solid tumors are encouraging, since to date clinical effectiveness of adoptive NK cell therapy is only observed in hematological malignancies. Therefore, more research on cetuximab unlocking the potential of adoptive NK cell therapy for solid tumors is warranted.

#### Chimeric Antigen Receptor (CAR)-Engineered NK Cells

A more recent and promising approach for adoptive NK cell therapy is the use of chimeric antigen receptor-engineered NK (CAR-NK) cells. These can be developed either through lenti-/retroviral transduction of primary adult NK cells or immortalized NK-92 cells to recognize a specific tumor antigen (134). CAR-NK cells have several advantages over CAR-T cells. First, they are more robust as they still maintain their intrinsic target cell recognition. Therefore, a reduction of the target CAR is less likely to be an effective tumor escape mechanism (135). Second, cytokines released by activated NK cells are less associated with the induction of a cytokine release syndrome (136, 137). Third, as NK cells do not clonally expand, the cytokine levels they release is found to be less sufficient to induce a cytokine release syndrome (138, 139). Fourth, NK cells are known to suppress graft-*versus*-host reactions which are induced by T cells due to strict HLA-matching (135, 136, 138).

While CAR-NK therapy research is developing at a rapid pace, combination treatments using CAR-NK together with already established treatments are still limited. Recently, combined treatment of a CRC mouse model with epithelial cell adhesion molecule (EpCAM)-CAR-NK-92 and regorafenib (a sorafenib-related multikinase inhibitor) achieved a synergistic tumor suppression than either treatment alone (140). The basis for this investigation was the observation that regorafenib could modulate the TIME through alteration of Fas and PD-L1 expression in CRC cell lines (140). Similarly, efficacy of cetuximab in HNSCC is also linked to its immunostimulatory activities which include downregulation of PD-L1 expression. Therefore, although not validated yet, this suggests that cetuximab combined with CAR-NK cells against a specific tumor antigen could alter the TIME towards tumor cell killing as a potentially promising treatment strategy. As a proof of concept, CAR-T cells transduced with CD32A or CD16 in combination with cetuximab, achieved a greater cytotoxic response and improved survival of a CRC mouse model bearing EGFR mutations compared to either treatment alone (141, 142). Taken together, although definitive evidence for this regimen is still missing, these early results support the potential strength of cetuximab-based dual-targeting CAR-NK therapy as an adoptive therapy.

A last consideration is that adoptive transfer of (un)modified NK cells in solid tumors is inferior compared to responses observed in hematological malignancies. The most evident cause for this discrepancy is the poor migration of infused NK cells inside the tumor. This may be caused by altered chemokine receptors following *ex vivo* activation. For example, CXCR2/3/4 are important chemokine receptors on immune cells that facilitate migration towards CXCL9/10/12-expressing tumor cells (143–146). Loss of CXCR2/3 following *ex vivo* activation prevented NK cells from migrating towards B16 melanoma tumors (147). Therefore, more recent expansion protocols such as the one described by Somanchi et al. (148) consider the chemokine repertoire in order to achieve efficient expansions of specific NK cell phenotypes that may provide a better invasion in the tumor.

### Targeting Negative Immune Checkpoint Molecules Prevents Immune Escape

**Figure 4:**
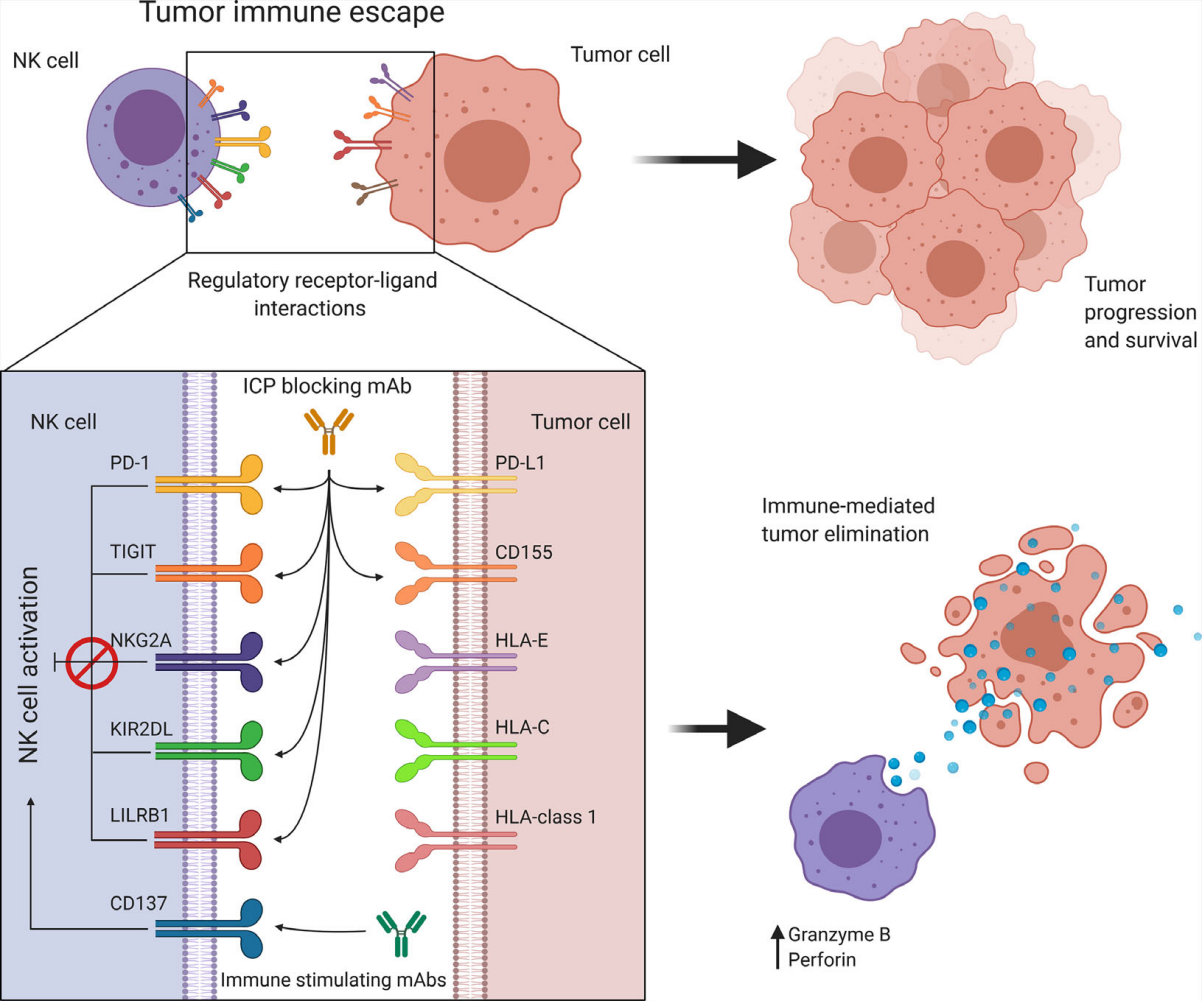
Targeting immune regulatory molecules improves immune effector function against cancer. NK cell activity is regulated by a balance between immune activating and inhibiting interactions. Cancer promotes immune checkpoint expression to suppress NK cell activation allowing tumor immune escape and progression. Antibody-based immunotherapies suppress inhibitory signaling or further activate costimulatory signals to restore and enhance NK cell activity. HLA, Human leukocyte antigen; KIR, Killer cell immunoglobulin-like receptor; LILRB1, Leukocyte immunoglobulin-like receptor B1; NK, Natural killer; NKG2A, Natural killer group 2A; PD-1, Programmed cell death protein 1; PD-L1, Programmed cell death ligand 1; TIGIT, T cell immunoreceptor with Ig and ITIM domains.

Discovery of immune checkpoint blockade has played a pivotal role towards integration of immunotherapy into clinical cancer treatment. While initial immune checkpoint inhibitors, such as anti-CTLA-4 (ipilimumab) and anti-PD-1 (pembrolizumab) have focused on reversing the suppressed state of cytotoxic T cells (149), current research is expanding this to other cell types, including NK cells (**Figure 4**). This expanded research also brought with it an increasing number of molecules that are being investigated as possible immune checkpoints and an endless possibility for combinations with checkpoint inhibitors to achieve greater responses.

#### Programmed Cell Death Protein 1 (PD-1) Pathway

The PD-1/PD-L1 axis has become one of the most studied pathways in cancer immunotherapy, with promising results guiding the approval of several inhibitors (150, 151). Interestingly, early investigations of PD-1 expression on NK cells found 25% of healthy individuals to have PD-1^+^ NK cells which correlated well with prior human cytomegalovirus infections (152). This prompted the idea that PD-1 expression on NK cells is a result of activation rather than exhaustion, which is the case for T cells following chronic stimulation (153). In cancer patients, peripheral blood NK cells are often found to be PD-1 positive (154–156) and intratumoral NK cells often express high levels of PD-1 (40, 156).

Interestingly, PD-1^+^ NK cells were found to have downregulated CD16 expression and induce PD-L1 expression on tumor cells *via* IFNγ secretion, thus possibly inhibiting ADCC induction (157). However, inhibition of EGFR-signaling *via* cetuximab is known to interrupt INF-γ signaling and prevent PD-L1 upregulation on tumor cells (117). Thus, combining cetuximab with a PD-1 inhibitor could be viewed as a valuable strategy to prevent CD16 downregulation and PD-1/PD-L1-axis mediated silencing of ADCC. A study in HNSCC found an increased number of PD-1^+^ NK cells in patients, which correlated with a diminished NK cell activity, as observed by a downregulated expression of CD16, CD107a and GZMB. In addition, PD-L1 expression correlated with a lack of response to cetuximab alone. Administration of cetuximab in combination with the anti-PD-1 mAb nivolumab successfully reversed NK cell diminishment and enhanced cetuximab-mediated ADCC *in vitro* (157). Early results from a phase I trial in HNSCC patients also reported an increased objective response rate compared to either treatment alone (158). Currently, several trials investigating this combination are ongoing, with preliminary results indicating potentially synergistic effects in advanced solid tumors (159, 160).

#### T Cell Immunoreceptor With Ig and ITIM Domains (TIGIT) Pathway

Recent years have seen a growing interest in the TIGIT signaling pathway due to its complex immunomodulatory role. Similar to the B7/CD28/CTLA-4 pathway, the TIGIT axis consists of a network of inhibitory receptors (TIGIT, CD96 and CD112R) that compete with the activating receptor (DNAM-1/CD226) for their shared ligands (CD111/NECTIN1, CD112/NECTIN2, CD113/NECTIN3, CD155/PVR) (161, 162). In contrast to DNAM-1, only marginal TIGIT expression is observed on resting NK and T cells while stimulation and tumor infiltration showed upregulated TIGIT expression (161). As a stimulatory receptor, DNAM-1 signaling induces pro-inflammatory cytokine secretion and enhances cytotoxic activity of NK cells. Meanwhile, TIGIT induces an anti-inflammatory, non-proliferative and non-cytotoxic profile in NK cells (163).

Targeting of TIGIT is still in early development but positive early (pre)clinical investigations have enabled further clinical investigations. Interestingly, *in vitro* co-culture and *in vivo* transgenic HNSCC mice models were able to restore the cytotoxic effects of T and NK cells following anti-TIGIT treatment (164). Initial clinical studies in solid tumors demonstrated strong antitumor activity as a single agent (163, 165), that could be further improved when combined with anti-PD1 mAb (NCT03119428, NCT02794571). Furthermore, disruption of the TIGIT/CD155 interaction can also beneficially impact the TIME, in particular by incapacitating myeloid-derived suppressor cells and depleting Tregs. Although not investigated yet, this observation suggests the possible combination of anti-TIGIT-mAbs with cetuximab, thereby reducing the suppressive action of Tregs and targeting specific tumor antigens.

Alternatively to TIGIT, CD155 (PVR), has been suggested as a potential target due to its greater affinity towards TIGIT compared to DNAM-1 and its frequent overexpression in solid tumors (166, 167). However, clinical trials of CD155 are still scarce and preclinical investigations of CD155 in combination with cetuximab are limited as well. However, one study in CRC cell lines reported an improvement of cetuximab-mediated ADCC following effective signaling of DNAM-1/CD155. Blocking this interaction abrogated this effect entirely (168). The same effect was observed by blocking NKG2D/MICA/B signaling. A possible reason for the limited progress in CD155 targeting might be that CD155 inhibition disrupts both TIGIT and DNAM-1 signaling, therefore potentially robbing NK cells from activating signals. However, this concern is not completely warranted, as CD155 under normal circumstances has a greater affinity towards inhibitory receptors, thus preferentially signaling *via* TIGIT even in the presence of DNAM-1 (169). Lastly, administration of anti-CD155 also showed upregulation of DNAM-1 on peripheral blood lymphocytes. As CD155 is not the only ligand capable of binding DNAM-1, this interaction could potentially shift the balance towards increased antitumor immunity (170).

Altogether, this suggests that strategies targeting the TIGIT-axis could reverse immune inhibition through reduced inhibitory signaling and that combinations with cetuximab could enhance ADCC, resulting in an enhanced antitumor response (167).

#### C-Type Lectin NK Cell Group 2 (NKG2) Subfamily Pathway

Another ITIM-containing signaling pathway expressed on NK and T cells is the NKG2A-HLA-E interaction. Although NKG2A is expressed on a low number of peripheral NK cells, both antigen and cytokine stimulation upregulate its expression (171, 172). While binding of NKG2A to HLA-E is known to inhibit NK cell responses, ovarian cancer cell lines that were treated with the anti-NKG2A mAb monalizumab showed profound antitumor responses and significantly improved cetuximab-mediated ADCC (173, 174). Moreover, monalizumab combined with cetuximab was tested in a phase II trial with recurrent and metastatic HNSCC patients showing promising improvements with an easily manageable safety profile similar to either treatment alone (173). Another trial, where monalizumab was combined with durvalumab (anti-PD-1 mAb) in CRC showed encouraging activity as well (175). Meanwhile, a phase III randomized trial in HNSCC has been announced for this combination (176). Therefore, an anti-NKG2A mAb could be a promising checkpoint inhibitor to enhance antitumor immunity of both T and NK cells.

#### Killer-Cell Immunoglobulin-Like Receptor (KIR) Pathway

KIRs play a major role in regulating NK cell activity through various inhibitory and activating receptors and are most frequently found on intratumoral CD56^dim^ NK cells (29, 171). Similar to IFNγ, the inhibition of EGFR can increase HLA-C expression through STAT-1 signaling (26, 177). Thus, this could potentially limit NK cell responses through an increased interaction of KIRs with HLA-C. The use of mAbs, such as lirilumab (IPH2102), targeting KIR2DL-1/-2/-3, can mimic the mismatch of KIR with HLA-C and prevent inhibitory signaling. Indeed, various (pre-)clinical reports have described an improved NK cell cytotoxicity following lirilumab treatment (178–180). Furthermore, combination of lirilumab with an anti-CD20 mAb enhanced ADCC against lymphoma cells *in vitro* and *in vivo* (180). Similarly, lirilumab in combination with cetuximab induced a significantly higher cytotoxic response against HNSCC cell lines in co-culture experiments (149). Hence, despite the lack of extensive literature, investigations of lirilumab in combination with cetuximab suggest that could generate clinical benefit and therefore warrant further investigation. Importantly however, long-term treatment with lirilumab may also hold some drawbacks. To fully develop into functionally mature cells, NK cells undergo a process of ‘education’ whereby their level of exposure and interaction to ‘self’ antigens with inhibitory receptors will determine their responsiveness in cases where these antigens are missing (181). Therefore, it is thought that persistent inhibition of KIRs could, besides stimulating the activity of mature NK cells, impede the development of new, functionally competent NK cells (178). In this regard, future clinical trials will have to resolve the optimal scheduling of blockade of inhibitory receptors.

#### Leukocyte Immunoglobulin-Like Receptor B (LILRB) Pathway

Similar to KIRs although far less understood, leukocyte immunoglobulin‐like receptors (LILRs)can regulate immune activity through ligation with MHC class I molecules. However, in contrast to the extensive KIR repertoire being expressed, NK cells predominantly express LILRB1 (182, 183). Interestingly, LILRB1 expression negatively correlated with cetuximab-induced ADCC against breast cancer patients (184). Furthermore, blocking LILRB1 increased both natural cytotoxicity as well as cetuximab-mediated ADCC, especially when both NK cells and cancer cells expressed LILRB1. Interestingly, LILRB1 expression and cetuximab-mediated ADCC were positively correlated in this context, indicating a greater inhibition at higher LILRB1 expression levels. However, LILRB1 research is still limited and factors impacting the regulation of LILRB1 expression should be the focus of future research to assess the potential for clinical implementation of this combination.

### Immune Agonists Allow Positive Immune Checkpoint Therapy

Since NK cells are dependent on a balance between positive and negative signals, negative signaling from immune checkpoints is counterbalanced by immune stimulatory molecules that positively enhance antitumor responses. Early attempts of developing potent agonist therapies were met with tremendous clinical toxicities due to selection of CD28, a constitutively expressed ‘second signal’ receptor on T cells, as a target. Theralizumab, despite the promising preclinical results, induced severe cytokine release syndrome with a high proportion of multiple organ failure in a phase I trial (185). Therefore, cautioned and rational selection of stimulatory molecules is essential to prevent non-discriminatory immune stimulation. Current approaches mostly comprise of selecting inducible targets following stimulation or maturation, rather than constitutive expression by immune cells (186).

#### Tumor Necrosis Factor Receptor Superfamily Member 9 (CD137/TNFRSF9)

Of interest for the context of this review is the molecule CD137 (4-1BB), expressed on various immune cells following pro-inflammatory stimuli (187). Signaling through CD137 delivers an enhanced tumor-selective cytotoxicity and IFNγ secretion (188). Interestingly, CD137 agonistic mAbs are classified as either strong or weak agonistic Abs. The difference is that strong agonistic Abs (Urelumab) can activate 4-1BB without FcγR-mediated crosslinking, while the weak agonistic Abs (Utomilumab) require FcγR-mediated crosslinking to activate 4-1BB. However, the effects of both classes can still be enhanced through separate FcγR-crosslinking (189). In this regard, although urelumab alone in a breast cancer xenograft model had no effect on tumor size, combined treatment with trastuzumab enhanced trastuzumab-mediated killing significantly (190). Furthermore, urelumab together with cetuximab greatly improved survival of HNSCC patients and elevated DC maturation and T cell cross-presentation together with an increased cytokine secretion (185, 186). Interestingly, TINK but not peripheral blood NK cells substantially increased CD137 expression following treatment with cetuximab. Both urelumab and cetuximab alone also upregulated anti-apoptotic proteins (Bcl-xL and Bcl-2) in NK cells, suggesting an improved survival of activated NK cells, that was further increased following combination treatment (186). These results suggest that urelumab could indeed be combined with cetuximab to enhance immune activity. However, the early clinical observations remain to be investigated in larger cohorts and various tumor types to develop a stronger support for this notion.

#### Pattern Recognition Receptors (PRR)

A critical role in pathogen recognition is carried out by toll-like receptors (TLRs). As part of the innate immunity, TLRs play a vital role in activating immune responses as well. This is achieved through recognition of pathogen- or damage-associated molecular patterns (PAMPs and DAMPs) expressed by microorganisms or released from damaged or dying cells (191). While a total of 11 TLRs have been identified, TLR7/8 are of particular interest in cancer research due to their direct immune stimulatory effect and simultaneous ablation of Treg function (192, 193). Therefore, stimulation of TLR7/8 could be an interesting treatment in tumors that are highly infiltrated with effector and suppressive immune cells. Stimulation through TLR7/8 could potentially polarize the TIME towards tumor killing by producing Th1-polarizing cytokines such as TNF-α, IFNγ and IL-12 (192). In this regard, the use of the TLR8 agonist motolimod, increased peripheral blood mononuclear cell cytotoxicity against HNSCC cell lines, together with a higher production of inflammatory cytokines and chemokines by DCs, monocytes and NK cells (194). Additionally, ADCC was enhanced through combination with cetuximab as well (194, 195), showing a possible way to effectively activate innate and adaptive anticancer immune responses. A phase I trial in HNSCC reported encouraging antitumor activity without dose limiting toxicities when motolimod was combined with cetuximab. Furthermore, increases in plasma cytokine levels and in frequency and activation of circulating NK cells were observed as well (196). Currently, this combination is being further investigated in a phase II randomized trial (NCT01836029) of chemotherapy plus cetuximab in combination with motolimod in patients with recurrent or metastatic HNSCC.

As part of the PRR family, the stimulator of interferon genes (STING) DNA sensing pathway forms an important part of the innate immunity, as it recognizes cytoplasmic DNA through Cyclic GMP-AMP synthase (cGAS), gamma-interferon-inducible protein 16 (IFI16) and probable ATP-dependent RNA helicase (DDX41) (197). Therefore, STING also recognizes tumor-DNA and induces downstream signaling of NF-κB and interferon regulatory factor 3 (IRF-3). This results in the induction IFNs and inflammatory cytokines such as TNF-α, IL-1β and IL-6 (198). However, STING can also induce mitochondrial apoptosis through Bcl-2-associated X protein (Bax) induction (199). Therefore, the use of STING agonists to induce an inflammatory microenvironment and induce direct tumor apoptosis may be a valuable treatment. However, some reports suggest that STING may play a dual role in cancer, potentially promoting tumor growth in tumors with low antigenicity (200). Therefore, combined treatment of STING agonists with other treatments may achieve a good clinical outcome. Interestingly, EGFR was found to affect IRF-3 phosphorylation, suggesting a possibility for cetuximab to be combined with a STING agonist to enhance IRF-3 signaling and thereby lead to an enhanced antitumor response (201). Indeed, STING activation enhanced cetuximab-mediated ADCC of NK cells against HNSCC cell lines and promoted NK : DC crosstalk, suggesting an important role of STING in effective antitumor immunity (202). A phase I trial of the STING agonist dimethylxanthone acetic acid (DMXAA) (murine STING agonist) plus carboplatin, paclitaxel and cetuximab only demonstrated limited activity due to limited binding to human STING (NCT01031212). However, other clinical trials using human counterparts of STING agonists have provided clinical evidence for its therapeutic effectiveness. However, as no phase III trials have been registered yet, it remains to be seen what the exact clinical benefit of this combination will be. Regardless, the accumulated data so far point towards integration of immune-stimulatory molecules into standardized treatment regimens to induce clinically exploitable systemic responses.

### Cytokine-Based Immune Potentiation

Cytokines form a group of small short-lived polypeptides that are involved in growth, differentiation and pro- and/or anti-inflammatory signals depending on the cell type. Although usually secreted in response to a defined stimulus, cytokines such as IL-7, required for immune cell homeostasis, can be constitutively expressed as well (203). Additionally, tumor cells can also secrete cytokines, mostly towards the establishment of an immunosuppressive TIME. Exogeneous administration of immunostimulatory cytokines has long been utilized in several lines of immunological investigations as a means of re-establishing the functionality of the immune system.

#### Interleukin-2 (IL-2)

Characterization of immunosuppressive factors and their involvement in tumor immune escape mechanisms has prompted researchers to reverse these impaired cytotoxic interactions through implementation of immunostimulatory cytokines. A study in HSNCC patients displayed elevated plasma levels TGF-β1 and soluble MHC I chain-related peptide A (sMICA) to diminish NKG2D expression, TNF-α and IFNγ release by NK cells, suppressing their antitumor responses (204). Interestingly, although NKG2D was downregulated due to high sMICA/TGF-β1 levels, CD16 expression and cetuximab-induced ADCC remained unaltered (204). Furthermore, IL-2 stimulation improved ADCC of sMICA inhibited NK cells resulting in a restored TNF-α and IFNγ secretion (204). Similarly, several other investigations in solid tumors have reported a significantly enhanced antitumor activity with tolerable safety profiles and improved ADCC following combined treatment with IL-2 and cetuximab (133, 205, 206). However, IL-2 administration in patients also causes expansion of FoxP3^+^ Tregs, which highly express the IL-2α receptor (207). Tumor types with relatively low intratumoral Tregs could potentially still benefit from this combination, as shown by the studies above (133, 208, 209). In contrast, tumors such as HNSCC and melanoma have been characterized as the most Treg infiltrated tumor types, making the use of IL-2 in combination with cetuximab less attractive (29, 210). Therefore, the makeup of the TIME is an important consideration that must be evaluated on a tumor type basis for this combination to be of value.

#### Interleukin-12 (IL-12)

One of the first alternatives to IL-2 was IL-12, a cytokine produced by DCs and macrophages. IL-12 has anti-bacterial and anti-angiogenic effects and enhances the immune response to Ab-coated tumor cells (211). Stimulation of NK cells with IL-12 leads to secretion of IFNγ and TNF-α, as well as increased levels of chemokines such as MIP-1α, IL-8 and RANTES, further stimulating the infiltration of CD8^+^ T cells into the tumor. Additionally, IL-12 increases IL-2α expression by NK cells, further enhancing NK cell activity in response to endogenous IL-2 (212). A phase I/II trial of heavily pretreated HNSCC patients investigated the combination of IL-12 with cetuximab and achieved stable disease in 69% of patients, with prolonged PFS. Additionally, ADCC responses were increased together with higher levels of IFNγ, CXCL10 and TNF-α secretion (213). IL-12 was also able to suppress Treg function through downregulation of FoxP3 (207, 214). Thus, in addition to stimulating NK cells, IL-12 administration may also reverse immune tolerance and creates a less suppressive TIME, enhancing antitumor immunity.

#### Interleukin-15 (IL-15)

IL-15 is a cytokine produced primarily by monocytes and macrophages and stimulates various NK and T cell functions (215). Similar to IL-2, stimulation with IL-15 is able to enhance the antitumor effects of NK cells against various tumor types and significantly increases cytokine and chemokine secretions (216, 217). Interestingly, besides upregulation of CD16, NKG2D and IFNγ, levels of NKp30 and NKp46 on NK cells of CRC patients were restored following IL-15 stimulation (216). However, IL-15 based therapies face some limitations as well, including a short serum half-life, narrowing down the therapeutic window, and the requirement for IL-15 receptor α-chain (IL-15Rα)-binding prior to activating effector cells, which limits the therapeutic application (218, 219). More recently, the genetically modified IL-15 compound ALT-803, consisting of IL-15 plus the IL-15Rα fused to the Fc portion of IgG1, has been developed in order to address the limitations of IL-15-based therapies. As a result, ALT-803 has higher biological activity and a longer serum half-life compared with free IL-15. Consistently, ALT-803 was able to enhance the ADCC response following cetuximab treatment in HNSCC cell lines to a level similar to or better than IL-15. In mice, while single-agent treatment partially reduced tumor growth, co-administration of cetuximab with ALT-803 showed complete tumor regression and increased secretion of IFNγ, RANTES and IL-8 (218). Early clinical trials with ALT-803 alone have reported promising efficacy and activity, showing an increased expansion of NK and CD8^+^ T cells (220). Interestingly, combination of ALT-803 with rituximab, another ADCC inducing mAb, gave similar results as ALT-803 plus cetuximab, thus supporting the exploration of ALT-803 to enhance cetuximab therapy (221).

#### Interleukin-21 (IL-21)

IL-21 belongs to the IL-2 family of cytokines, based on the shared cytokine receptor γ chain (γ_c_). In comparison to IL-2 and IL-15, single-agent treatment with IL-21 was shown to be the most potent antitumor cytokine with longer lasting responses and clearing mice from tumors in settings where both IL-2 and IL-15 only showed limited effect (222). Additionally, IL-21 stimulation was also shown to increase levels of IL-2α in addition to IFNγ, perforin and GZMB (223). Interestingly, the combination of IL-21 with cetuximab was also able to enhance the ability of NK cells to recognize and eliminate cetuximab-coated tumor cells (223–225). Clinical trials using IL-21 in combination with cetuximab confirm preclinical findings, reporting increased cytokine secretion, enhanced ADCC and achieving stable disease in patients with different tumor types (225, 226).

Although we have discussed the drawback involved in IL-2 treatment regarding Treg expansion, cytokines also have faced criticism as a potential immunotherapeutic approach, due to additional limitations. These include the relatively short serum half-life, requiring careful exploration of clinical doses that could otherwise lead to severe toxic responses (227). Furthermore, IL-2 and IL-12 induce vascular leaking due to alterations in vascular permeability, which is only minimally present with IL-15 and IL-21 treatment (228, 229). These limitations lie at the basis of the functional properties of cytokines. However, they have not stopped researchers from investigating ways to enhance the effectiveness of cytokines through, for example, genetic engineering. The works of Skrombolas et al. and Berraondo et al. provide a detailed and comprehensive review regarding these strategies (203, 208). Taken together, the combined use of cytokines with cetuximab as an ADCC inducing agent has the ability to restore/enhance cytolytic activity of NK cells. Future research likely will include genetically cytokine engineering or consider the use of cytokine cocktails. These could help provide optimal enhancement of NK cells and prevent the limitations involved with single cytokine administration.

### Combinations With Immunomodulatory Drugs

Although various novel compounds targeting tumor or immune antigens are in the developmental pipeline, another class of drugs that is of interest are the immunomodulatory drugs. These are a group of small molecules that were initially developed as treatment for other human diseases than cancer but were eventually recognized and exploited for their positive effects on the immune system.

#### Poly Adenosine Diphosphate (ADP)-Ribose Polymerase (PARP)

Cancer cells rely on DNA damage repair mechanisms to maintain their survival, making these repair pathways ideal targets for cancer treatment, e.g. poly Adenosine diphosphate (ADP)-ribose polymerase (PARP) (230). PARP enzymes act as DNA damage sensors when single-strand DNA breaks occur. Thus, PARP inhibition can severely inhibit cell survival, trigger cell cycle arrest and apoptosis through accumulation of DNA damage. Interestingly, PARP inhibition also activates the STING DNA sensing pathway, subsequently leading to production of type I IFN and pro-inflammatory cytokines, thus priming an antitumor immune response (231, 232). Therefore, the possibility to combine PARP inhibition with immunotherapy seems highly interesting.

EGFR inhibition with cetuximab diminishes DNA synthesis and double-strand break repair and therefore can increase tumor susceptibility to PARP inhibitors (233, 234). Indeed, combining cetuximab with PARP inhibitors significantly increased ADCC in both Breast cancer susceptibility protein (*BRCA*)-WT and -mutant cell lines (235). Clinically, a phase I study in locally advanced HNSCC patients demonstrated promising responses and tolerable toxicities (236), although results were confounded by continued smoking during treatment of non-responders (237). Thus, this combination warrants further study in a phase II setting to further investigate its effectiveness. The biggest risk involved with PARP inhibition is the potential to develop secondary myelodysplastic syndrome/acute myeloid leukemia due to impaired DNA damage repair. This was limited to patients that additionally received chemotherapy and had germline DNA repair deficiencies, further inducing DNA damage (238).

#### Thalidomide Derivatives

Despite the severe side effects observed with thalidomide in the 1960s, its mechanisms of action have revealed immunomodulatory and anti-angiogenic activity. Analogues such as lenalidomide and pomalidomide are more potent immunomodulators and have fewer side effects. Lenalidomide has been approved for treatment of multiple hematological malignancies, as it is known to activate cytokine production, regulate T cell co-stimulation and augment NK cell cytotoxicity (239, 240). Lenalidomide is believed to enhance NK cell functionality in an indirect manner, mainly related to the release of IL-2 by other immune cells (240). Lenalidomide also enhanced ADCC following combination with several IgG1 mAbs, including cetuximab (168, 241). So far, the suggested mechanisms report that this enhancement is likely the result of an increased CD16 expression (168) and partly attributable to an increased presence of IL-2 and/or IL-12 cytokines secreted by T cells or other immune cells (242). On the other hand, lenalidomide-enhanced ADCC was abrogated through blocking of either DNAM-1/CD155 interactions or NKG2D with its ligands, indicating that optimal enhancement of ADCC requires interactions of DNAM-1 and NKG2D (168). Clinical trials investigating the combination of lenalidomide with cetuximab are currently in phase I/II and report a well-tolerated treatment with promising clinical activity in patients with CRC and HNSCC. Moreover, a dose-dependent increase in NK cytotoxic activity was demonstrated, with increasing doses of lenalidomide. This was associated with a significantly increased ADCC activity and an increased number of CD8^+^ T cells and circulating NK cells (243, 244).

Thus, immunomodulating agents such as PARP inhibitors or lenalidomide combined with EGFR-directed therapies show promising preclinical and early clinical results but remain to be investigated in more detail.

## Conclusion & Future Perspectives

Although cetuximab is an established therapeutic agent in HNSCC and CRC, a major roadblock in achieving durable responses is the onset of therapeutic resistance. In contrast, immunotherapy can achieve long-lasting disease control, but only in a small percentage of patients. The TIME plays an important role in cancer‐specific drug responses. The recent approval of pembrolizumab as a first-line treatment in HNSCC has sparked an increased interest in the modulation of immune responses to further improve survival of HNSCC patients (245). As increasing evidence points towards immune responses as a major determinant of mAb efficacy, it becomes increasingly difficult not to endorse the rationale of combination therapies. The earliest attempts, for example using IL-2, have indeed enhanced effector functions at the cost of stimulating immunosuppressive cells as well. Current approaches minimize unwanted effects by rational selection of targets such as IL-15. We previously showed that healthy NK cells may overcome cetuximab resistance *in vitro* (68). However, overcoming clinical resistance to cetuximab may require additional immunotherapies to harness the full potential of NK cells. In this review we have discussed several approaches to augment cetuximab-mediated ADCC against solid tumors.

The majority of approaches discussed in this review focus on manipulation of cell surface receptors and cytokines to enhance NK cell activity. These promising early results warrant further research, as there is a window for improvement and a requirement to tailor these strategies to various tumor types. For example, as HNSCC is marked with the highest infiltration of NK cells, effective treatment should focus on enhancing NK cell activity, by reducing inhibitory signaling or increasing activating signals. In contrast, CRC only shows marginal NK cell infiltration and thus the primary objective should be to lure NK cells inside the tumor, either through adoptive transfer or through increased homing. A better understanding of cancer-specific immune interactions will undoubtedly yield stronger scientific and clinical endeavors.

The current era of genomic, transcriptomic and immune profiling analysis will likely improve the tailoring of single-agent or combination therapies towards patient populations, thus entering an era of precision immunotherapy. Key components towards the success of future trials are considerations towards incorporating ADCC, intratumoral persistence and trafficking of NK cells. In this regard, given the clinical results summarized in this review are mostly still under phase I/II investigation, we anticipate future studies to confirm that cetuximab in combination with immune checkpoint inhibitors synergistically enhances the innate and adaptive antitumor immune responses. There are currently at least 109 active trials investigating cetuximab in a combination regimen with various other treatments (clinicaltrials.gov). Of these, at least 19 trials are investigating combinations with immunotherapeutic modalities discussed above (**Table 2**). The potential of cetuximab-based NK cell immunotherapy looks promising and we foresee that NK cells will become appreciated as a natural component in the fight against cancer.

**Table 2:** ADCC-mediating IgG1 therapeutic antibodies.

Antibody (Trade name)	Company	Approval FDA/EMA*	Indication	Target	IgG1 type	Fc modification	Reference
**Unmodified Fc Abs**							
Alemtuzumab (Campath)	Ilex Pharmaceuticals	2013	MS	CD52	Humanized	/	(1)
Avelumab (Bavencio)	Merck KGaA and Pfizer	2017	MCC, UC, RCC	PD-L1	Human	/	(2)
Cetuximab (Erbitux)	Bristol-Myers Squibb	2004	HNSCC, CRC	EGFR	Chimeric	/	(3)
Dinutuximab (Unituxin)	United Therapeutics	2015	NB	GD2	Chimeric	/	(4)
Ipilimumab (Yervoy)	Bristol-Myers Squibb	2011	MEL, RCC,	CTLA-4	Human	/	(5)
Necitumumab (Portrazza)	Eli Lilly and Company	2015/2016	NSCLC	EGFR	Human	/	(6)
Ofatumumab (Arzerra)	Genmab	2009/2010	CLL	CD20	Human	/	(7)
Pertuzumab (Perjeta)	Genentech	2012/2013	BCA	HER2/neu	Humanized	/	(8)
Rituximab (Rituxan)	Genentech	1997/1998	NHL, CLL	CD20	Chimeric	/	(9)
Trastuzumab (Herceptin)	Genentech	1998/2000	BCA, GC	HER2/neu	Humanized	/	(10)
**Fc modified Abs**							
Imgatuzumab	Genentech	/	HNSCC	EGFR	Humanized	Reduced fucosylation	(11)
Margetuximab (Margenza)	MacroGenics	2020/2018	BCA	HER2/neu	Chimeric	Enhanced FcγRIII binding (F^243^L; R^292^P; Y^300^L; V^305^I; P^396^L)	(12)
Mogamulizumab (Poteligeo)	Kyowa Hakko Kirin	2018	CTCL	CCR4	Humanized	Afucosylated	(13)
Obinutuzumab (Gazyva)	Roche	2013/2014	CLL, FL	CD20	Humanized	Afucosylated	(14)
Tafasitamab (Monjuvi)	MorphoSys	2020	DLBCL	CD19	Humanized	Enhanced FcγRIII binding (S^239^D; I^332^E)	(15)
Tomuzotuximab (CetuGEX)	Glycotope	/	NSCLC, CRC, HNSCC, GC	EGFR	Chimeric	Afucosylated	(16)

BCA, Breast cancer; CCR4, Chemokine receptor 4; CLL, Chronic lymphocytic leukemia; CRC, Colorectal cancer; CTCL, Cutaneous T-cell lymphoma; CTLA-4, Cytotoxic T-lymphocyte-associated protein 4; DLBCL, Diffuse large B-cell lymphoma; EGFR, Epidermal growth factor receptor; EMA, European Medicines Agency; FDA, Food and Drug Administration; FL, Follicular lymphoma; GC, Gastric cancer; GD2, Disialoganglioside; HER, Epidermal growth factor receptor 2; HNSCC, Head and neck squamous cell carcinoma; I.V., Intravenously; MCC, Merkel cell carcinoma; MEL, Melanoma; MS, Multiple sclerosis; NB, Neuroblastoma; NSCLC, Non-small cell lung cancer; RCC, Renal cell carcinoma; UC, urothelial carcinoma.*Approval by FDA and EMA within the same year if only a single date is given.

Although we exclusively discussed cetuximab as the primary ADCC-inducing agent in this review, a large portion of these applications could be applied to other IgG1 mAbs (**Table 3**). In this regard, we believe the NK cell-based discussed approaches could also be of interest for other cancer indications employing ADCC-inducing mAbs. Moreover, growing research focuses on the development of engineered mAbs that display enhanced ADCC. These modifications involve altering the mAb Fc portion to increase binding affinity to FcγRIIIa *via* site-directed mutagenesis, editing Fc domain glycosylation and/or removing Fc domain fucosylation. Various Fc-engineered mAbs have shown improved responses compared to unmodified counterparts and have gained approval for clinical use (**Table 3**).

**Table 3:** Active clinical trials evaluating cetuximab in combination with NK cell stimulating immunotherapies.

Clinical trial ID	Study phase	Estimated patients	Initial registration	Indication	Treatment	Primary endpoint	Status
**Adoptive NK cell therapy**							
NCT03319459	I	100	2018	Advanced Solid Tumors	FATE-NK100	DLT	Active, not recruiting
					FATE-NK100 + trastuzumab		
					FATE-NK100 + cetuximab		
NCT04872634	I/II	24	2021	LA/M NSCLC	SNK01 (low/high dose) + gemcitabin e	MTD, AE	Recruiting
					SNK01 (low/high) + Cetuximab + gemcitabin e		
							
**Cytokines**							
NCT01468896	I/II	23	2011	R/M HNSCC	Recombinant interleukin-12 + cetuximab	DLT, OR	Active, not recruiting
NCT02627274	I	134	2015	Solid tumors	RO6874281	DLT, MTD, OBD	Active, not recruiting
					RO6874281 + Trastuzumab		
					RO6874281 + cetuximab		
NCT04616196	I/II	78	2020	R/M HNSCC & CRC	Dose Escalation of NKTR-255 + cetuximab	AE, ORR	Recruiting
					Dose expansion of NKTR-255 + cetuximab		
**EGFR-TKI**							
NCT02716311	II	118	2016	EGFR mutant NSCLC	Afatinib	TTF	Active, not recruiting
					Afatinib + cetuximab		
NCT02979977	II	50	2016	Advanced HNSCC	Afatinib + cetuximab	ORR	Recruiting
NCT03727724	II	37	2018	NSCLC	Afatinib + cetuximab	DCR	Recruiting
NCT04820023	I/II	90	2021	Advance d NSCLC	BBT-176	AE, DLT, ORR	Recruiti ng
					BBT-176 + cetuximab		
**NKG2A**							
NCT02643550	I/II	143	2015	R/M HNSCC	Monalizumab + cetuximab	DLT, ORR	Active, not recruitin g
					monalizumab + cetuximab + anti-PD(L)1		
NCT04349267	I/II	308	2020	Advanced Solid	BMS-986315	AE	Recruiti
				Tumors	BMS-986315 + nivolumab		ng
					BMS-986315 + cetuximab		
NCT04590963	III	600	2020	R/M HNSCC	Monalizumab + cetuximab	OS	Recruiting
					Placebo + cetuximab		
**PD-1/PD-L1**							
NCT02999087	III	707	2016	LA HNSCC	CRT	PFS	Active, not recruiting
					Cetuximab + RT + avelumab		
NCT03174405	II	43	2017	mCRC	Avelumab + cetuximab + FOLFOX	PFS	Active, not recruiting
NCT03494322	II	130	2018	R/M HNSCC	Avelumab	DLT, DCR	not recruiting
					Avelumab + cetuximab		
NCT03498378	I	24	2018	R/M HNSCC	Avelumab + cetuximab + palbociclib	MTD	Recruiting
NCT03608046	II	59	2018	mCRC	Avelumab + cetuximab + irinotecan	ORR	Recruiting
NCT03944252	II	54	2018	LA & R/M SCCAC	Avelumab	ORR	Active, not recruiting
					Avelumab + cetuximab		
NCT04561336	II	77	2018	RAS-WT mCRC	Avelumab + cetuximab	OS	Active, not recruiting

AE, Adverse events, CR, Complete response, CRT, Chemoradiotherapy, CSCC, Cutaneous squamous cell cancer, DCR, Disease control rate, DLT, Dose limiting toxicity, ESqCC, Esophageal squamous cell carcinoma, HNSCC, head and neck squamous cell carcinoma, LA, Locally advanced, mCRC, Metastatic colorectal carcinoma, MTD, Maximum tolerated dose, OBD, Optimal biological dose, OR, Objective response, ORR, Objective response rate, OS, Overall survival, PFS, Progression free survival, R/M, Recurrent and metastatic, RT, radiotherapy, SCCAC, Squamous cell anal carcinoma, TTF, Time to treatment failure, WT, Wild-type.

Implementation of any combination treatment requires a strong consideration for potential AEs. Biomarkers for EGFR targeting include EGFR gene amplifications and mutations, but also downstream sarcoma viral oncogene (Ras), PI3K and PTEN activities as well (102, 246). As downstream oncogenic signaling can affect the TIME, it is important to consider immunological biomarkers as well. Besides PD-L1 expression on tumors, factors such as PD-L1 on immune cells and co-expression of other inhibitory checkpoints may affect the response to PD-1 targeting (247). Furthermore, consideration of tumor immune infiltration, proportion of immune cell phenotypes and tumor mutational burden have proven to be a better representation for the effectiveness of immunotherapies in solid tumors (40, 248–250).

Importantly, despite the overall success of immune checkpoint inhibitors in various tumor types, meta-analyses often show severe treatment-related AEs that are associated with tumor response. In most patients, these AEs are related to overstimulation of immune reactivity. However, the severity of AEs is dependent on the used inhibitor. For example, CTLA-4 inhibitors have a higher risk of treatment-related AEs compared to PD-1/PD-L1 inhibitors (251). A possible solution might be targeting several biological pathways to induce longer-lasting responses. Interestingly, while the use of dual checkpoint inhibition or combination with TKI increased dose-sensitivity with higher risk of toxicity, mAb combinations, including cetuximab, that aim to elicit higher ADCC responses could be given at their recommended phase II doses without greatly increasing toxicities (252). Nevertheless, future research should always consider the potential for increased AEs in any combination strategy and dose-escalation schemes are greatly useful in that regard.

The next couple of years will undoubtedly bring a more in-depth understanding of the TIME together with the next generation of targets for anticancer treatment. This will allow us to rationally design better combination therapies in order to achieve the most optimal long-term effectiveness. In this era, we believe that cetuximab and many other ADCC-capable mAbs will remain valuable components, as it becomes clear that mAbs can add great benefit to both conventional and immunotherapies. As NK cell activation depends on a balance of stimulatory and inhibitory signals, the combinations that involve stimulation of NK cells through ADCC, together with suppression of inhibitory signals or the attraction of NK cells are of particular interest. As these combinations are currently under (pre)clinical investigation, the knowledge they provide regarding valuable biomarkers will soon guide the next generation of clinical trial measurements and ultimately lead to higher-quality treatments that will provide the most effective benefit to the patient.

## Author Contributions

HB, JDW, IDP, JBV, and AW conceived the presented idea. The writing of the presented work was supervised by JW, IP, and AW. The original draft was written by HB and reviewed & edited by HB, JW, IP, HZ, MP, JV, FL, and AW. All authors contributed to the article and approved the submitted version. All figures were created using BioRender.com

## Funding

The research was funded by Kom op tegen Kanker with grant number OZ7886 (Stand up to Cancer), the Flemish cancer society.

## Conflict of Interest

The authors declare that the research was conducted in the absence of any commercial or financial relationships that could be construed as a potential conflict of interest.

## Publisher’s Note

All claims expressed in this article are solely those of the authors and do not necessarily represent those of their affiliated organizations, or those of the publisher, the editors and the reviewers. Any product that may be evaluated in this article, or claim that may be made by its manufacturer, is not guaranteed or endorsed by the publisher.

## Abbreviations

ADCC, Antibody-dependent cellular cytotoxicity; ADP, Adenosine diphosphate; AE, Adverse events; ATP, Adenosine triphosphate; BRCA, Breast cancer susceptibility protein; CAR, Chimeric antigen receptor; CRC, Colorectal cancer; CTLA-4, Cytotoxic T-lymphocyte-associated protein 4; DAMP, Damage-associated molecular patterns; DC, Dendritic cell; DDX41, Probable ATP-dependent RNA helicase; DMXAA, Dimethylxanthone acetic acid; EGFR, Epidermal growth factor receptor; EMA, European Medicines Agency; EpCAM, Epithelial cell adhesion molecule; FAS, First apoptosis signal; FDA, Food and drug administration; cGAS, Cyclic GMP-AMP synthase; IFI16, Gamma-interferon-inducible protein Ifi-16; GZMB, Granzyme B; HLA, Human leukocyte antigen; HNSCC, Head and neck squamous cell cancer; IFNγ, Interferon gamma; IL, Interleukins; IRF, Interferon regulatory factor 3; ITAM, Immunoreceptor tyrosine-based activation motif; ITIM, Immunoreceptor tyrosine-based inhibitory motif; KIR, Killer-cell immunoglobulin-like receptors; LAG-3, Lymphocyte-activation gene 3; LILR, Leukocyte immunoglobulin-like receptor B; MHC, Major histocompatibility complex; MIC, MHC class I polypeptide–related sequence A/B; NCR, Natural cytotoxicity receptor; NK, Natural killer; NSCLC, Non-small cell lung cancer; NT5E, 5’-nucleotidase; OS, Overall survival; PAMP, pathogen-associated molecular patterns; PARP, Poly adenosine diphosphate-ribose polymerase; PD-1, Programmed cell death protein; PFS, Progression free survival; PRR, Pattern recognition receptors; PTEN, Phosphatase and tensin homolog; STING, Stimulator of interferon genes; TGF, Transforming growth factor; TIGIT, T cell immunoreceptor with Ig and ITIM domains; TIME, tumor immune microenvironment; TINK, tumor-infiltrating NK cells; TKI, Tyrosine kinase inhibitor;TLR, Toll-like receptors; TME, Tumor microenvironment; TNFR, Tumor necrosis factor-related apoptosis-inducing ligand receptor; TNFRSF9, Tumor necrosis factor receptor superfamily member 9; TRAIL, TNF-related apoptosis-inducing ligand; ULBP, UL16-binding protein; VEGF, Vascular endothelial growth factor; XIAP, X-linked inhibitor of apoptosis protein; YINM, Tyrosine-based signaling motif; ZAP70, Zeta-chain associated protein kinase.

## References

[1] Derakhshani A Rostami Z Taefehshokr S Safarpour H Astamal RV Taefehshokr N. An Overview of the Oncogenic Signaling Pathways in Different Types of Cancers. Preprints.org (2020).

[2] Mahipal A Kothari N Gupta S. Epidermal Growth Factor Receptor Inhibitors: Coming of Age. Cancer Control (2014) 21(1):74–9. 10.1177/107327481402100111 24357745

[3] Carpenter G Cohen S. Epidermal Growth Factor. Annu Rev Biochem (1979) 48:193–216. 10.1146/annurev.bi.48.070179.001205 382984

[4] Leemans CR Braakhuis BJ Brakenhoff RH. The Molecular Biology of Head and Neck Cancer. Nat Rev Cancer (2011) 11(1):9–22. 10.1038/nrc2982 21160525

[5] Oda K Matsuoka Y Funahashi A Kitano H. A Comprehensive Pathway Map of Epidermal Growth Factor Receptor Signaling. Mol Syst Biol (2005) 1. 10.1038/msb4100014 PMC1681468 16729045

[6] Kimura H Sakai K Arao T Shimoyama T Tamura T Nishio K. Antibody-Dependent Cellular Cytotoxicity of Cetuximab Against Tumor Cells With Wild-Type or Mutant Epidermal Growth Factor Receptor. Cancer Sci (2007) 98(8):1275–80. 10.1111/j.1349-7006.2007.00510.x PMC11159318 17498200

[7] Harari PM. Epidermal Growth Factor Receptor Inhibition Strategies in Oncology. Endocr Relat Cancer (2004) 11(4):689–708. 10.1677/erc.1.00600 15613446

[8] Saxena B Sundaram ST Walton W Patel I Kuo P Khan S. Differentiation Between the EGFR Antibodies Necitumumab, Cetuximab, and Panitumumab: *In Vitro* Biological and Binding Activities. J Clin Oncol (2011) 29(15_suppl):e13030–0. 10.1200/jco.2011.29.15_suppl.e13030

[9] Bagchi A Haidar JN Eastman SW Vieth M Topper M Iacolina MD. Molecular Basis for Necitumumab Inhibition of EGFR Variants Associated With Acquired Cetuximab Resistance. Mol Cancer Ther (2018) 17(2):521–31. 10.1158/1535-7163.MCT-17-0575 PMC5925748 29158469

[10] Trivedi S Srivastava RM Concha-Benavente F Ferrone S Garcia-Bates TM Li J. Anti-EGFR Targeted Monoclonal Antibody Isotype Influences Antitumor Cellular Immunity in Head and Neck Cancer Patients. Clin Cancer Res (2016) 22(21):5229–37. 10.1158/1078-0432.CCR-15-2971 PMC5093040 27217441

[11] Tay RY Wong R Hawkes EA. Treatment of Metastatic Colorectal Cancer: Focus on Panitumumab. Cancer Manag Res (2015) 7:189–98. 10.2147/CMAR.S71821 PMC4484697 26150735

[12] Price T Kim TW Li J Cascinu S Ruff P Suresh AS. Final Results and Outcomes by Prior Bevacizumab Exposure, Skin Toxicity, and Hypomagnesaemia From ASPECCT: Randomized Phase 3 non-Inferiority Study of Panitumumab *Versus* Cetuximab in Chemorefractory Wild-Type KRAS Exon 2 Metastatic Colorectal Cancer. Eur J Cancer (2016) 68:51–9. 10.1016/j.ejca.2016.08.010 27716478

[13] Sugimoto N Sakai D Tamura T Hara H Nishina T Esaki T. Randomized Phase II Study of Panitumumab (Pmab) + Irinotecan (CPT-11) *Versus* Cetuximab (Cmab) + CPT-11 in Patients (Pts) With KRAS Wild-Type (WT) Metastatic Colorectal Cancer (Mcrc) After Fluoropyrimidine (FU), CPT-11, and Oxaliplatin (L-OHP) Failure: WJOG6510G. J Clin Oncol (2017) 35(4_suppl):661–1. 10.1200/JCO.2017.35.4_suppl.661

[14] Giralt J Trigo J Nuyts S Ozsahin M Skladowski K Hatoum G. Panitumumab Plus Radiotherapy *Versus* Chemoradiotherapy in Patients With Unresected, Locally Advanced Squamous-Cell Carcinoma of the Head and Neck (CONCERT-2): A Randomised, Controlled, Open-Label Phase 2 Trial. Lancet Oncol (2015) 16(2):221–32. 10.1016/S1470-2045(14)71200-8 25596659

[15] Siu LL Waldron JN Chen BE Winquist E Wright JR Nabid A. Phase III Randomized Trial of Standard Fractionation Radiotherapy (SFX) With Concurrent Cisplatin (CIS) *Versus* Accelerated Fractionation Radiotherapy (AFX) With Panitumumab (Pmab) in Patients (Pts) With Locoregionally Advanced Squamous Cell Carcinoma of the Head and Neck (LA-SCCHN): NCIC Clinical Trials Group HN.6 Trial. J Clin Oncol (2015) 33(15_suppl):6000–0. 10.1200/jco.2015.33.15_suppl.6000

[16] Bonner JA Harari PM Giralt J Azarnia N Shin DM Cohen RB. Radiotherapy Plus Cetuximab for Squamous-Cell Carcinoma of the Head and Neck. N Engl J Med (2006) 354(6):567–78. 10.1056/NEJMoa053422 16467544

[17] Vermorken JB Remenar E Hitt R Kawecki A Rottey S Knierim L. Platinum-Based Chemotherapy (CT) Plus Cetuximab in Recurrent or Metastatic Squamous Cell Carcinoma of the Head and Neck Cancer (R/M-SCCHN): 5-Year Follow-Up Data for the Extreme Trial. J Clin Oncol (2014) 32(15_suppl):6021–1. 10.1200/jco.2014.32.15_suppl.6021

[18] Shurin MR Naiditch H Gutkin DW Umansky V Shurin GV. Chemoimmunomodulation: Immune Regulation by the Antineoplastic Chemotherapeutic Agents. Curr Med Chem (2012) 19(12):1792–803. 10.2174/092986712800099785 22414087

[19] Shurin MR. Dual Role of Immunomodulation by Anticancer Chemotherapy. Nat Med (2013) 19(1):20–2. 10.1038/nm.3045 PMC4045109 23296003

[20] Stagg J Andre F Loi S. Immunomodulation *via* Chemotherapy and Targeted Therapy: A New Paradigm in Breast Cancer Therapy? Breast Care (Basel) (2012) 7(4):267–72. 10.1159/000342166 PMC3515790 23904828

[21] Dunn GP Old LJ Schreiber RD. The Immunobiology of Cancer Immunosurveillance and Immunoediting. Immunity (2004) 21(2):137–48. 10.1016/j.immuni.2004.07.017 15308095

[22] Muenst S Laubli H Soysal SD Zippelius A Tzankov A Hoeller S. The Immune System and Cancer Evasion Strategies: Therapeutic Concepts. J Intern Med (2016) 279(6):541–62. 10.1111/joim.12470 26748421

[23] Beatty GL Gladney WL. Immune Escape Mechanisms as a Guide for Cancer Immunotherapy. Clin Cancer Res (2015) 21(4):687–92. 10.1158/1078-0432.CCR-14-1860 PMC4334715 25501578

[24] Bauernhofer T Kuss I Henderson B Baum AS Whiteside TL. Preferential Apoptosis of CD56dim Natural Killer Cell Subset in Patients With Cancer. Eur J Immunol (2003) 33(1):119–24. 10.1002/immu.200390014 12594840

[25] Accomando WP Wiencke JK Houseman EA Butler RA Zheng S Nelson HH. Decreased NK Cells in Patients With Head and Neck Cancer Determined in Archival DNA. Clin Cancer Res (2012) 18(22):6147–54. 10.1158/1078-0432.CCR-12-1008 PMC3500449 23014525

[26] Lopez-Albaitero A Nayak JV Ogino T Machandia A Gooding W DeLeo AB. Role of Antigen-Processing Machinery in the in Vitro Resistance of Squamous Cell Carcinoma of the Head and Neck Cells to Recognition by CTL. J Immunol (2006) 176(6):3402–9. 10.4049/jimmunol.176.6.3402 16517708

[27] Almand B Resser JR Lindman B Nadaf S Clark JI Kwon ED. Clinical Significance of Defective Dendritic Cell Differentiation in Cancer. Clin Cancer Res (2000) 6(5):1755–66. 10815894

[28] Markovic SN Kumar AB. Therapeutic Targets of FDA-Approved Immunotherapies in Oncology. In: Dong H Markovic SN, editors. The Basics of Cancer Immunotherapy. Cham: Springer International Publishing (2018). p. 21–37.

[29] Mandal R Senbabaoglu Y Desrichard A Havel JJ Dalin MG Riaz N. The Head and Neck Cancer Immune Landscape and its Immunotherapeutic Implications. JCI Insight (2016) 1(17):e89829. 10.1172/jci.insight.89829 27777979 PMC5070962

[30] Diaz L Le D Yoshino T Andre T Koshiji M Zhang Y. KEYNOTE-177: Randomized Phase III Study of Pembrolizumab *Versus* Investigator-Choice Chemotherapy for Mismatch Repair-Deficient or Microsatellite Instability-High Metastatic Colorectal Carcinoma . J Clin Oncol (2017) 35:TPS815–5. 10.1200/JCO.2017.35.4_suppl.TPS815

[31] Pollack BP Sapkota B Cartee TV. Epidermal Growth Factor Receptor Inhibition Augments the Expression of MHC Class I and II Genes. Clin Cancer Res (2011) 17(13):4400–13. 10.1158/1078-0432.CCR-10-3283 21586626

[32] Concha-Benavente F Srivastava RM Trivedi S Lei Y Chandran U Seethala RR. Identification of the Cell-Intrinsic and -Extrinsic Pathways Downstream of EGFR and Ifngamma That Induce PD-L1 Expression in Head and Neck Cancer. Cancer Res (2016) 76(5):1031–43. 10.1158/0008-5472.CAN-15-2001 PMC4775348 26676749

[33] Wang T Niu G Kortylewski M Burdelya L Shain K Zhang S. Regulation of the Innate and Adaptive Immune Responses by Stat-3 Signaling in Tumor Cells. Nat Med (2004) 10(1):48–54. 10.1038/nm976 14702634

[34] Concha-Benavente F Ferris RL. Reversing EGFR Mediated Immunoescape by Targeted Monoclonal Antibody Therapy. Front Pharmacol (2017) 8:332. 10.3389/fphar.2017.00332 28611673 PMC5447743

[35] Wang H-C Chan L-P Cho S-F. Targeting the Immune Microenvironment in the Treatment of Head and Neck Squamous Cell Carcinoma. Front Oncol (1084) 2019:9. 10.3389/fonc.2019.01084 PMC6803444 31681613

[36] Peltanova B Raudenska M Masarik M. Effect of Tumor Microenvironment on Pathogenesis of the Head and Neck Squamous Cell Carcinoma: A Systematic Review. Mol Cancer (2019) 18(1):63. 10.1186/s12943-019-0983-5 30927923 PMC6441173

[37] Garcia-Lora A Algarra I Garrido F. MHC Class I Antigens, Immune Surveillance, and Tumor Immune Escape. J Cell Physiol (2003) 195(3):346–55. 10.1002/jcp.10290 12704644

[38] Nguyen N Bellile E Thomas D McHugh J Rozek L Virani S. Tumor Infiltrating Lymphocytes and Survival in Patients With Head and Neck Squamous Cell Carcinoma. Head Neck (2016) 38(7):1074–84. 10.1002/hed.24406 PMC4900934 26879675

[39] Habif G Crinier A Andre P Vivier E Narni-Mancinelli E. Targeting Natural Killer Cells in Solid Tumors. Cell Mol Immunol (2019) 16(5):415–22. 10.1038/s41423-019-0224-2 PMC6474204 30911118

[40] Wagner S Wittekindt C Reuschenbach M Hennig B Thevarajah M Wurdemann N. CD56-Positive Lymphocyte Infiltration in Relation to Human Papillomavirus Association and Prognostic Significance in Oropharyngeal Squamous Cell Carcinoma. Int J Cancer (2016) 138(9):2263–73. 10.1002/ijc.29962 26662627

[41] Monteverde M Milano G Strola G Maffi M Lattanzio L Vivenza D. The Relevance of ADCC for EGFR Targeting: A Review of the Literature and a Clinically-Applicable Method of Assessment in Patients. Crit Rev Oncol Hematol (2015) 95(2):179–90. 10.1016/j.critrevonc.2015.02.014 25819749

[42] Taylor RJ Saloura V Jain A Goloubeva O Wong S Kronsberg S. Ex Vivo Antibody-Dependent Cellular Cytotoxicity Inducibility Predicts Efficacy of Cetuximab. Cancer Immunol Res (2015) 3(5):567–74. 10.1158/2326-6066.CIR-14-0188 PMC4681575 25769300

[43] Lattanzio L Denaro N Vivenza D Varamo C Strola G Fortunato M. Elevated Basal Antibody-Dependent Cell-Mediated Cytotoxicity (ADCC) and High Epidermal Growth Factor Receptor (EGFR) Expression Predict Favourable Outcome in Patients With Locally Advanced Head and Neck Cancer Treated With Cetuximab and Radiotherapy. Cancer Immunol Immunother (2017) 66(5):573–9. 10.1007/s00262-017-1960-8 PMC11029535 28197666

[44] Lanier LL Phillips JH Hackett J Jr Tutt M Kumar V. Natural Killer Cells: Definition of a Cell Type Rather Than a Function. J Immunol (1986) 137(9):2735–9. 3489775

[45] Marcus A David H. Raulet, *Evidence for Natural Killer Cell Memory* . Curr Biol (2013) 23(17):R817–20. 10.1016/j.cub.2013.07.015 PMC3831844 24028966

[46] Westermann J Pabst R. Distribution of Lymphocyte Subsets and Natural Killer Cells in the Human Body. Clin Investig (1992) 70(7):539–44. 10.1007/BF00184787 1392422

[47] Gibson SE Swerdlow SH Felgar RE. Natural Killer Cell Subsets and Natural Killer–Like T-Cell Populations in Benign and Neoplastic B-Cell Proliferations Vary Based on Clinicopathologic Features. Hum Pathol (2011) 42(5):679–87. 10.1016/j.humpath.2010.07.023 PMC3677227 21292303

[48] Levi I Amsalem H Nissan A Darash-Yahana M Peretz T Mandelboim 0. Characterization of Tumor Infiltrating Natural Killer Cell Subset. Oncotarget (2015) 6(15):13835–43. 10.18632/oncotarget.3453 PMC4537053 26079948

[49] Stabile H Fionda C Gismondi A Santoni A. Role of Distinct Natural Killer Cell Subsets in Anticancer Response. Front Immunol (2017) 8:293. 10.3389/fimmu.2017.00293 28360915 PMC5352654

[50] Carotta S. Targeting NK Cells for Anticancer Immunotherapy: Clinical and Preclinical Approaches. Front Immunol (2016) 7:152. 10.3389/fimmu.2016.00152 27148271 PMC4838611

[51] Ljunggren H-G Kärre K. In Search of the ‘Missing Self’: MHC Molecules and NK Cell Recognition. Immunol Today (1990) 11:237–44. 10.1016/0167-5699(90)90097-S 2201309

[52] Langers I Renoux V Thiry M Delvenne P Jacobs N. Natural Killer Cells: Role in Local Tumor Growth and Metastasis. (2012) 6:73–82. 10.2147/BTT.S23976 PMC3333822 22532775

[53] Long EO Kim HS Liu D Peterson ME Rajagopalan S. Controlling Natural Killer Cell Responses: Integration of Signals for Activation and Inhibition. Annu Rev Immunol (2013) 31:227–58. 10.1146/annurev-immunol-020711-075005 PMC3868343 23516982

[54] Mace EM Orange JS. New Views of the Human NK Cell Immunological Synapse: Recent Advances Enabled by Super- and High-Resolution Imaging Techniques. Front Immunol (2012) 3:421. 10.3389/fimmu.2012.00421 23316204 PMC3540402

[55] Lux A Yu X Scanlan CN Nimmerjahn F. Impact of Immune Complex Size and Glycosylation on Igg Binding to Human Fcγrs. J Immunol (2013) p:1200501. 10.4049/jimmunol.1200501 23509345

[56] Bournazos S Gupta A Ravetch JV. The Role of Igg Fc Receptors in Antibody-Dependent Enhancement. Nat Rev Immunol (2020) 20(10):633–43. 10.1038/s41577-020-00410-0 PMC7418887 32782358

[57] Hsu HT Carisey AF Orange JS. Measurement of Lytic Granule Convergence After Formation of an NK Cell Immunological Synapse. Methods Mol Biol (2017) 1584:497–515. 10.1007/978-1-4939-6881-7_31 28255722 PMC5861262

[58] Green DR Llambi F. Cell Death Signaling. Cold Spring Harbor Perspect Biol (2015) 7(12):a006080. 10.1101/cshperspect.a006080 PMC4665079 26626938

[59] Mirandola P Ponti C Gobbi G Sponzilli I Vaccarezza M Cocco L. Activated Human NK and CD8+ T Cells Express Both TNF-Related Apoptosis-Inducing Ligand (TRAIL) and TRAIL Receptors But are Resistant to TRAIL-Mediated Cytotoxicity. Blood (2004) 104(8):2418–24. 10.1182/blood-2004-04-1294 15205263

[60] Campbell KS Purdy AK. Structure/Function of Human Killer Cell Immunoglobulin-Like Receptors: Lessons From Polymorphisms, Evolution, Crystal Structures and Mutations. Immunology (2011) 132(3):315–25. 10.1111/j.1365-2567.2010.03398.x PMC3044898 21214544

[61] Borrego F Masilamani M Kabat J Sanni TB Coligan JE. The Cell Biology of the Human Natural Killer Cell CD94/NKG2A Inhibitory Receptor. Mol Immunol (2005) 42(4):485–8. 10.1016/j.molimm.2004.07.031 15607803

[62] Kim N Kim HS. Targeting Checkpoint Receptors and Molecules for Therapeutic Modulation of Natural Killer Cells. Front Immunol (2018) 9:2041. 10.3389/fimmu.2018.02041 30250471 PMC6139314

[63] Zhou XM Li WQ Wu YH Han L Cao XG Yang XM. Intrinsic Expression of Immune Checkpoint Molecule TIGIT Could Help Tumor Growth in Vivo by Suppressing the Function of NK and CD8(+) T Cells. Front Immunol (2018) 9:2821. 10.3389/fimmu.2018.02821 30555485 PMC6281988

[64] Le Bert N Gasser S. Advances in NKG2D Ligand Recognition and Responses by NK Cells. Immunol Cell Biol (2014) 92(3):230–6. 10.1038/icb.2013.111 24445601

[65] Kruse PH Matta J Ugolini S Vivier E. Natural Cytotoxicity Receptors and Their Ligands. Immunol Cell Biol (2014) 92(3):221–9. 10.1038/icb.2013.98 24366519

[66] Vivier E Nunes JA Vely F. Natural Killer Cell Signaling Pathways? Science (2004) 306(5701):517–9. 10.1126/science.1103478 15567854

[67] Tomasello E Blery M Vely F Vivier F. Signaling Pathways Engaged by NK Cell Receptors: Double Concerto for Activating Receptors, Inhibitory Receptors and NK Cells? Semin Immunol (2000) 12(2):139–47. 10.1006/smim.2000.0216 10764622

[68] Wang W Erbe AK Hank JA Morris ZS Sondel PM. NK Cell-Mediated Antibody-Dependent Cellular Cytotoxicity in Cancer Immunotherapy. Front Immunol (2015) 6:368. 10.3389/fimmu.2015.00368 26284063 PMC4515552

[69] Kono K Takahashi A Ichihara F Sugai H Fujii H Matsumoto Y. Impaired Antibody-Dependent Cellular Cytotoxicity Mediated by Herceptin in Patients With Gastric Cancer. Cancer Res (2002) 62(20):5813–7. 12384543

[70] Naidu B. Monoclonal Antibodies With ADCC and CDC Enhancement for Therapy. Int J Pharma Bio Sci (2013) 4:B588–99.

[71] Patel D Guo X Ng S Melchior M Balderes P Burtrum D. Igg Isotype, Glycosylation, and EGFR Expression Determine the Induction of Antibody-Dependent Cellular Cytotoxicity in Vitro by Cetuximab. Hum Antibodies (2010) 19(4):89–99. 10.3233/HAB-2010-0232 21178280

[72] Yang X Zhang X Mortenson ED Radkevich-Brown O Wang Y Fu YX. Cetuximab-Mediated Tumor Regression Depends on Innate and Adaptive Immune Responses. Mol Ther (2013) 21(1):91–100. 10.1038/mt.2012.184 22990672 PMC3538305

[73] Ahmed M Pan DW Davis ME. Lack of in Vivo Antibody Dependent Cellular Cytotoxicity With Antibody Containing Gold Nanoparticles. Bioconjugate Chem (2015) 26(5):812–6. 10.1021/acs.bioconjchem.5b00139 PMC4445771 25879583

[74] García-Foncillas J Sunakawa Y Aderka D Wainberg Z Ronga P Witzler P. Distinguishing Features of Cetuximab and Panitumumab in Colorectal Cancer and Other Solid Tumors. Front Oncol (2019) 9(849). 10.3389/fonc.2019.00849 PMC6763619 31616627

[75] Veluchamy JP Spanholtz J Tordoir M Thijssen VL Heideman DA Verheul HM. Combination of NK Cells and Cetuximab to Enhance Anti-Tumor Responses in RAS Mutant Metastatic Colorectal Cancer. PloS One (2016) 11(6):e0157830. 10.1371/journal.pone.0157830 27314237 PMC4912059

[76] Taylor RJ Chan SL Wood A Voskens CJ Wolf JS Lin W. Fcgammariiia Polymorphisms and Cetuximab Induced Cytotoxicity in Squamous Cell Carcinoma of the Head and Neck. Cancer Immunol Immunother (2009) 58(7):997–1006. 10.1007/s00262-008-0613-3 18979096 PMC11030953

[77] Fujii R Schlom J Hodge JW. A Potential Therapy for Chordoma *via* Antibody-Dependent Cell-Mediated Cytotoxicity Employing NK or High-Affinity NK Cells in Combination With Cetuximab. J Neurosurg (2018) 128(5):1419–27. 10.3171/2017.1.JNS162610 PMC6459012 28753113

[78] Lopez-Albaitero A Lee SC Morgan S Grandis JR Gooding WE Ferrone S. Role of Polymorphic Fc Gamma Receptor Iiia and EGFR Expression Level in Cetuximab Mediated, NK Cell Dependent in Vitro Cytotoxicity of Head and Neck Squamous Cell Carcinoma Cells. Cancer Immunol Immunother (2009) 58(11):1853–64. 10.1007/s00262-009-0697-4 PMC3426289 19319529

[79] Nakamura H Tamaki S Yagyuu T Yamakawa N Hatake K Kirita T. Relationship Between EGFR Expression in Oral Cancer Cell Lines and Cetuximab Antibody-Dependent Cell-Mediated Cytotoxicity. Anticancer Res (2019) 39(3):1275–82. 10.21873/anticanres.13238 30842158

[80] Strauss L Bergmann C Szczepanski M Gooding W Johnson JT Whiteside TL. A Unique Subset of CD4+CD25highFoxp3+ T Cells Secreting Interleukin-10 and Transforming Growth Factor-Beta1 Mediates Suppression in the Tumor Microenvironment. Clin Cancer Res (2007) 13(15 Pt 1):4345–54. 10.1158/1078-0432.CCR-07-0472 17671115

[81] Jie HB Srivastava RM Argiris A Bauman JE Kane LP Ferris RL. Increased PD-1(+) and TIM-3(+) Tils During Cetuximab Therapy Inversely Correlate With Response in Head and Neck Cancer Patients. Cancer Immunol Res (2017) 5(5):408–16. 10.1158/2326-6066.CIR-16-0333 PMC5497750 28408386

[82] Baysal H De Pauw I Zaryouh H De Waele J Peeters M Pauwels P. Cetuximab-Induced Natural Killer Cell Cytotoxicity in Head and Neck Squamous Cell Carcinoma Cell Lines: Investigation of the Role of Cetuximab Sensitivity and HPV Status. Br J Cancer (2020). 10.1038/s41416-020-0934-3 PMC7462851 32541873

[83] Xu JM Wang Y Wang YL Wang Y Liu T Ni M. PIK3CA Mutations Contribute to Acquired Cetuximab Resistance in Patients With Metastatic Colorectal Cancer. Clin Cancer Res (2017) 23(16):4602–16. 10.1158/1078-0432.CCR-16-2738 PMC5559326 28424201

[84] Eze N Lee J-W Yang D-H Zhu F Neumeister V Sandoval-Schaefer T. PTEN Loss is Associated With Resistance to Cetuximab in Patients With Head and Neck Squamous Cell Carcinoma. Oral Oncol (2019) 91:69–78. 10.1016/j.oraloncology.2019.02.026 30926065 PMC6855599

[85] Kondo N Tsukuda M Taguchi T Nakazaki K Sakakibara A Takahashi H. Gene Status of Head and Neck Squamous Cell Carcinoma Cell Lines and Cetuximab-Mediated Biological Activities. Cancer Sci (2011) 102(9):1717–23. 10.1111/j.1349-7006.2011.01999.x 21631644

[86] Wang GQ Wieckowski E Goldstein LA Gastman BR Rabinovitz A Gambotto A. Resistance to Granzyme B-Mediated Cytochrome C Release in Bak-Deficient Cells. J Exp Med (2001) 194(9):1325–37. 10.1084/jem.194.9.1325 PMC2195982 11696597

[87] Medema JP de Jong J Peltenburg LT Verdegaal EM Gorter A Bres SA. Blockade of the Granzyme B/Perforin Pathway Through Overexpression of the Serine Protease Inhibitor PI-9/SPI-6 Constitutes a Mechanism for Immune Escape by Tumors . Proc Natl Acad Sci U.S.A. (2001) 98(20):11515–20. 10.1073/pnas.201398198 PMC58761 11562487

[88] van Houdt IS Oudejans JJ van den Eertwegh AJM Baars A Vos W Bladergroen BA. Expression of the Apoptosis Inhibitor Protease Inhibitor 9 Predicts Clinical Outcome in Vaccinated Patients With Stage III and IV Melanoma. Clin Cancer Res (2005) 11(17):6400–7. 10.1158/1078-0432.CCR-05-0306 16144945

[89] Evans MK Sauer SJ Nath S Robinson TJ Morse MA Devi GR. X-Linked Inhibitor of Apoptosis Protein Mediates Tumor Cell Resistance to Antibody-Dependent Cellular Cytotoxicity. Cell Death Dis (2016) 7:e2073. 10.1038/cddis.2015.412 26821068 PMC4816185

[90] Baginska J Viry E Berchem G Poli A Noman MZ van Moer K. Granzyme B Degradation by Autophagy Decreases Tumor Cell Susceptibility to Natural Killer-Mediated Lysis Under Hypoxia . Proc Natl Acad Sci U.S.A. (2013) 110(43):17450–5. 10.1073/pnas.1304790110 PMC3808626 24101526

[91] Pahl JHW Cerwenka A Ni J. Memory-Like NK Cells: Remembering a Previous Activation by Cytokines and NK Cell Receptors. Front Immunol (2018) 9:2796. 10.3389/fimmu.2018.02796 30546366 PMC6279934

[92] Kijima M Yamaguchi T Ishifune C Maekawa Y Koyanagi A Yagita H. Dendritic Cell-Mediated NK Cell Activation is Controlled by Jagged2–Notch Interaction. Proc Natl Acad Sci (2008) 105(19):7010–5. 10.1073/pnas.0709919105 PMC2383942 18458347

[93] Zwirner NW Domaica CI. Cytokine Regulation of Natural Killer Cell Effector Functions. Biofactors (2010) 36(4):274–88. 10.1002/biof.107 20623510

[94] Srivastava RM Lee SC Andrade Filho PA Lord CA Jie HB Davidson HC. Cetuximab-Activated Natural Killer and Dendritic Cells Collaborate to Trigger Tumor Antigen-Specific T-Cell Immunity in Head and Neck Cancer Patients. Clin Cancer Res (2013) 19(7):1858–72. 10.1158/1078-0432.CCR-12-2426 PMC3640274 23444227

[95] Vermorken JB Mesia R Rivera F Remenar E Kawecki A Rottey S. Platinum-Based Chemotherapy Plus Cetuximab in Head and Neck Cancer. N Engl J Med (2008) 359(11):1116–27. 10.1056/NEJMoa0802656 18784101

[96] Prewett MC Hooper AT Bassi R Ellis LM Waksal HW Hicklin DJ. Enhanced Antitumor Activity of Anti-Epidermal Growth Factor Receptor Monoclonal Antibody IMC-C225 in Combination With Irinotecan (CPT-11) Against Human Colorectal Tumor Xenografts. Clin Cancer Res (2002) 8(5):994–1003. 12006511

[97] Carvalho H Villar RC. Radiotherapy and Immune Response: The Systemic Effects of a Local Treatment . Clinics (Sao Paulo Brazil) (2018) 73(suppl 1):e557s–s. 10.6061/clinics/2018/e557s PMC6257057 30540123

[98] Bracci L Schiavoni G Sistigu A Belardelli F. Immune-Based Mechanisms of Cytotoxic Chemotherapy: Implications for the Design of Novel and Rationale-Based Combined Treatments Against Cancer. Cell Death Differentiation (2014) 21(1):15–25. 10.1038/cdd.2013.67 23787994 PMC3857622

[99] Korrer MJ Kim Y. Natural Killer Cells From Primary Human Head and Neck Squamous Cell Carcinomas Upregulate NKG2A. J Immunol (2017) 198(1 Supplement):130.18–8.

[100] Pries R Wulff S Kesselring R Borngen K Xie L Wollenberg B. Up-Regulation of NK Cell Function Against Head and Neck Cancer in Response to Ss-Isrna Requires TLR7. Int J Oncol (2008) 33(5):993–1000. 18949362

[101] Vitale M Cantoni C Pietra G Mingari MC Moretta L. Effect of Tumor Cells and Tumor Microenvironment on NK-Cell Function. Eur J Immunol (2014) 44(6):1582–92. 10.1002/eji.201344272 24777896

[102] Boeckx C Baay M Wouters A Specenier P Vermorken JB Peeters M. Anti-Epidermal Growth Factor Receptor Therapy in Head and Neck Squamous Cell Carcinoma: Focus on Potential Molecular Mechanisms of Drug Resistance. Oncologist (2013) 18(7):850–64. 10.1634/theoncologist.2013-0013 PMC3720640 23821327

[103] Braig F Kriegs M Voigtlaender M Habel B Grob T Biskup K. Cetuximab Resistance in Head and Neck Cancer is Mediated by EGFR-K521 Polymorphism. Cancer Res (2017) 77(5):1188–99. 10.1158/0008-5472.CAN-16-0754 28031227

[104] De Pauw I Lardon F Van den Bossche J Baysal H Fransen E Deschoolmeester V. Simultaneous Targeting of EGFR, HER2, and HER4 by Afatinib Overcomes Intrinsic and Acquired Cetuximab Resistance in Head and Neck Squamous Cell Carcinoma Cell Lines. Mol Oncol (2018) 12(6):830–54. 10.1002/1878-0261.12197 PMC5983215 29603584

[105] De Pauw I Lardon F Van den Bossche J Baysal H Pauwels P Peeters M. Overcoming Intrinsic and Acquired Cetuximab Resistance in RAS Wild-Type Colorectal Cancer: An in Vitro Study on the Expression of HER Receptors and the Potential of Afatinib. Cancers (Basel) (2019) 11(1). 10.3390/cancers11010098 PMC6357064 30650638

[106] Matar P Rojo F Cassia R Moreno-Bueno G Di Cosimo S Tabernero J. Combined Epidermal Growth Factor Receptor Targeting With the Tyrosine Kinase Inhibitor Gefitinib (ZD1839) and the Monoclonal Antibody Cetuximab (IMC-C225): Superiority Over Single-Agent Receptor Targeting. Clin Cancer Res (2004) 10(19):6487–501. 10.1158/1078-0432.CCR-04-0870 15475436

[107] Weickhardt AJ Price TJ Chong G Gebski V Pavlakis N Johns TG. Dual Targeting of the Epidermal Growth Factor Receptor Using the Combination of Cetuximab and Erlotinib: Preclinical Evaluation and Results of the Phase II DUX Study in Chemotherapy-Refractory, Advanced Colorectal Cancer. J Clin Oncol (2012) 30(13):1505–12. 10.1200/JCO.2011.38.6599 22412142

[108] Ramalingam S Forster J Naret C Evans T Sulecki M Lu H. Dual Inhibition of the Epidermal Growth Factor Receptor With Cetuximab, an Igg1 Monoclonal Antibody, and Gefitinib, a Tyrosine Kinase Inhibitor, in Patients With Refractory non-Small Cell Lung Cancer (NSCLC): A Phase I Study. J Thorac Oncol (2008) 3(3):258–64. 10.1097/JTO.0b013e3181653d1b 18317068

[109] Wheler JJ Tsimberidou AM Falchook GS Zinner RG Hong DS Fok JY. Combining Erlotinib and Cetuximab is Associated With Activity in Patients With non-Small Cell Lung Cancer (Including Squamous Cell Carcinomas) and Wild-Type EGFR or Resistant Mutations. Mol Cancer Ther (2013) 12(10):2167–75. 10.1158/1535-7163.MCT-12-1208 PMC4138057 23963360

[110] Gandara DR. Erlotinib and Cetuximab in Treating Patients With Advanced Solid Tumors With Emphasis on non-Small Cell Lung Cancer (2017). Available at: https://ClinicalTrials.gov/show/NCT00408499.

[111] Hasegawa H Yasuda H Hamamoto J Masuzawa K Tani T Nukaga S. Efficacy of Afatinib or Osimertinib Plus Cetuximab Combination Therapy for non-Small-Cell Lung Cancer With EGFR Exon 20 Insertion Mutations. Lung Cancer (2019) 127:146–52. 10.1016/j.lungcan.2018.11.039 30642543

[112] Gibbons DL Byers LA. A HER 1-2 Punch: Dual EGFR Targeting Deals Resistance a Deadly Blow. Cancer Discovery (2014) 4(9):991–4. 10.1158/2159-8290.CD-14-0791 PMC4990388 25185188

[113] Kim H Kim SH Kim MJ Kim SJ Park SJ Chung JS. EGFR Inhibitors Enhanced the Susceptibility to NK Cell-Mediated Lysis of Lung Cancer Cells. J Immunother (2011) 34(4):372–81. 10.1097/CJI.0b013e31821b724a 21499124

[114] Bae JH Kim SJ Kim MJ Oh SO Chung JS Kim SH. Susceptibility to Natural Killer Cell-Mediated Lysis of Colon Cancer Cells is Enhanced by Treatment With Epidermal Growth Factor Receptor Inhibitors Through UL16-Binding Protein-1 Induction. Cancer Sci (2012) 103(1):7–16. 10.1111/j.1349-7006.2011.02109.x 21951556 PMC11164140

[115] Mei JZ Liu GJ Zhang XJ Zhao JZ Feng RT. Erlotinib Enhances the CIK Cell-Killing Sensitivity of Lung Adenocarcinoma A549 Cells. Genet Mol Res (2015) 14(2):3082–9. 10.4238/2015.April.10.18 25966072

[116] Marshall J Shapiro GI Uttenreuther-Fischer M Ould-Kaci M Stopfer P Gordon MS. Phase I Dose-Escalation Study of Afatinib, an Erbb Family Blocker, Plus Docetaxel in Patients With Advanced Cancer. Future Oncol (2013) 9(2):271–81. 10.2217/fon.12.195 23414476

[117] Im J Herrmann A Bernatchez C Haymaker C Molldrem J Hong W. Immune-Modulation by Epidermal Growth Factor Receptor Inhibitors: Implication on Anti-Tumor Immunity in Lung Cancer. PloS One (2016) 11:e0160004. 10.1371/journal.pone.0160004 27467256 PMC4965069

[118] Vantourout P Willcox C Turner A Swanson CM Haque Y Sobolev O. Immunological Visibility: Posttranscriptional Regulation of Human NKG2D Ligands by the EGF Receptor Pathway. Sci Transl Med (2014) 6(231):231ra49. 10.1126/scitranslmed.3007579 PMC3998197 24718859

[119] Cavazzoni A Alfieri RR Cretella D Saccani F Ampollini L Galetti M. Combined Use of Anti-Erbb Monoclonal Antibodies and Erlotinib Enhances Antibody-Dependent Cellular Cytotoxicity of Wild-Type Erlotinib-Sensitive NSCLC Cell Lines. Mol Cancer (2012) 11(1):91. 10.1186/1476-4598-11-91 23234355 PMC3577499

[120] Mallmann-Gottschalk N Sax Y Kimmig R Lang S Brandau S. EGFR-Specific Tyrosine Kinase Inhibitor Modifies NK Cell-Mediated Antitumoral Activity Against Ovarian Cancer Cells. Int J Mol Sci (2019) 20(19):4693. 10.3390/ijms20194693 PMC6801374 31546690

[121] Horn L Gettinger S Camidge DR Smit EF Janjigian YY Miller VA. Continued Use of Afatinib With the Addition of Cetuximab After Progression on Afatinib in Patients With EGFR Mutation-Positive non-Small-Cell Lung Cancer and Acquired Resistance to Gefitinib or Erlotinib. Lung Cancer (2017) 113:51–8. 10.1016/j.lungcan.2017.08.014 29110849

[122] Parkhurst MR Riley JP Dudley ME Rosenberg SA. Adoptive Transfer of Autologous Natural Killer Cells Leads to High Levels of Circulating Natural Killer Cells But Does Not Mediate Tumor Regression. Clin Cancer Res (2011) 17(19):6287–97. 10.1158/1078-0432.CCR-11-1347 PMC3186830 21844012

[123] Ishikawa T Okayama T Sakamoto N Ideno M Oka K Enoki T. Phase I Clinical Trial of Adoptive Transfer of Expanded Natural Killer Cells in Combination With Igg1 Antibody in Patients With Gastric or Colorectal Cancer. Int J Cancer (2018) 142(12):2599–609. 10.1002/ijc.31285 29388200

[124] Levy EM Roberti MP Mordoh J. Natural Killer Cells in Human Cancer: From Biological Functions to Clinical Applications. J BioMed Biotechnol (2011) 2011:676198. 10.1155/2011/676198 21541191 PMC3085499

[125] Heidenreich S Kröger N. Reduction of Relapse After Unrelated Donor Stem Cell Transplantation by KIR-Based Graft Selection. Front Immunol (2017) 8:41–1. 10.3389/fimmu.2017.00041 PMC5296332 28228753

[126] Ruggeri L Capanni M Urbani E Perruccio K Shlomchik WD Tosti A. Effectiveness of Donor Natural Killer Cell Alloreactivity in Mismatched Hematopoietic Transplants. Science (2002) 295(5562):2097–100. 10.1126/science.1068440 11896281

[127] Giebel S Locatelli F Lamparelli T Velardi A Davies S Frumento G. Survival Advantage With KIR Ligand Incompatibility in Hematopoietic Stem Cell Transplantation From Unrelated Donors. Blood (2003) 102(3):814–9. 10.1182/blood-2003-01-0091 12689936

[128] Sanchez-Martinez D Allende-Vega N Orecchioni S Talarico G Cornillon A Vo DN. Expansion of Allogeneic NK Cells With Efficient Antibody-Dependent Cell Cytotoxicity Against Multiple Tumors. Theranostics (2018) 8(14):3856–69. 10.7150/thno.25149 PMC6071536 30083264

[129] Arai S Meagher R Swearingen M Myint H Rich E Martinson J. Infusion of the Allogeneic Cell Line NK-92 in Patients With Advanced Renal Cell Cancer or Melanoma: A Phase I Trial. Cytotherapy (2008) 10(6):625–32. 10.1080/14653240802301872 18836917

[130] Friedman J Padget M Lee J Schlom J Hodge J Allen C. Direct and Antibody-Dependent Cell-Mediated Cytotoxicity of Head and Neck Squamous Cell Carcinoma Cells by High-Affinity Natural Killer Cells. Oral Oncol (2019) 90:38–44. 10.1016/j.oraloncology.2019.01.017 30846174 PMC6410731

[131] Jochems C Hodge JW Fantini M Fujii R Morillon YM 2nd Greiner JW. An NK Cell Line (Hank) Expressing High Levels of Granzyme and Engineered to Express the High Affinity CD16 Allele. Oncotarget (2016) 7(52):86359–73. 10.18632/oncotarget.13411 PMC5341330 27861156

[132] Liang S Lin M Niu L Xu K Wang X Liang Y. Cetuximab Combined With Natural Killer Cells Therapy: An Alternative to Chemoradiotherapy for Patients With Advanced non-Small Cell Lung Cancer (NSCLC). Am J Cancer Res (2018) 8(5):879–91. PMC5992505 29888109

[133] Adotevi O Godet Y Galaine J Lakkis Z Idirene I Certoux JM. *In Situ* Delivery of Allogeneic Natural Killer Cell (NK) Combined With Cetuximab in Liver Metastases of Gastrointestinal Carcinoma: A Phase I Clinical Trial. Oncoimmunology (2018) 7(5):e1424673. 10.1080/2162402X.2018.1424673 29721386 PMC5927529

[134] Barrett DM Singh N Porter DL Grupp SA June CH. Chimeric Antigen Receptor Therapy for Cancer. Annu Rev Med (2014) 65:333–47. 10.1146/annurev-med-060512-150254 PMC4120077 24274181

[135] Natural Killer Cells for Cancer Immunotherapy: A New CAR is Catching Up. EBioMedicine (2019) 39:1–2. 10.1016/j.ebiom.2019.01.018 30685121 PMC6355444

[136] Xie G Dong H Liang Y Ham JD Rizwan R Chen J. CAR-NK Cells: A Promising Cellular Immunotherapy for Cancer. EBioMedicine (2020) 59. 10.1016/j.ebiom.2020.102975 PMC7452675 32853984

[137] Thakar MS Kearl TJ Malarkannan S. Controlling Cytokine Release Syndrome to Harness the Full Potential of CAR-Based Cellular Therapy. Front Oncol (2019) 9:1529. 10.3389/fonc.2019.01529 32076597 PMC7006459

[138] Liu E Marin D Banerjee P Macapinlac HA Thompson P Basar R. Use of CAR-Transduced Natural Killer Cells in CD19-Positive Lymphoid Tumors. N Engl J Med (2020) 382(6):545–53. 10.1056/NEJMoa1910607 PMC7101242 32023374

[139] Islam R Pupovac A Evtimov V Boyd N Shu R Boyd R. Enhancing a Natural Killer: Modification of NK Cells for Cancer Immunotherapy. Cells (2021) 10(5):1058. 33946954 10.3390/cells10051058 PMC8146003

[140] Zhang Q Zhang H Ding J Liu H Li H Li H. Combination Therapy With Epcam-CAR-NK-92 Cells and Regorafenib Against Human Colorectal Cancer Models. J Immunol Research (2018) 2018:4263520. 10.1155/2018/4263520 30410941 PMC6205314

[141] Caratelli S Arriga R Sconocchia T Ottaviani A Lanzilli G Pastore D. *In Vitro* Elimination of Epidermal Growth Factor Receptor-Overexpressing Cancer Cells by CD32A-Chimeric Receptor T Cells in Combination With Cetuximab or Panitumumab. Int J Cancer (2020) 146(1):236–47. 10.1002/ijc.32663 PMC8711771 31479522

[142] Arriga R Caratelli S Lanzilli G Ottaviani A Cenciarelli C Sconocchia T. CD16-158-Valine Chimeric Receptor T Cells Overcome the Resistance of KRAS-Mutated Colorectal Carcinoma Cells to Cetuximab. Int J Cancer (2020) 146(9):2531–8. 10.1002/ijc.32618 PMC8711772 31396956

[143] Faber A Goessler UR Hoermann K Schultz JD Umbreit C Stern-Straeter J. SDF-1-CXCR4 Axis: Cell Trafficking in the Cancer Stem Cell Niche of Head and Neck Squamous Cell Carcinoma. Oncol Rep (2013) 29(6):2325–31. 10.3892/or.2013.2380 23563306

[144] Wolff HA Rolke D Rave-Fränk M Schirmer M Eicheler W Doerfler A. Analysis of Chemokine and Chemokine Receptor Expression in Squamous Cell Carcinoma of the Head and Neck (SCCHN) Cell Lines. Radiat Environ biophysics (2011) 50(1):145–54. 10.1007/s00411-010-0341-x PMC3040826 21085979

[145] Ding Q Lu P Xia Y Ding S Fan Y Li X. CXCL9: Evidence and Contradictions for its Role in Tumor Progression. Cancer Med (2016) 5(11):3246–59. 10.1002/cam4.934 PMC5119981 27726306

[146] Liu M Guo S Stiles JK. The Emerging Role of CXCL10 in Cancer (Review). Oncol Lett (2011) 2(4):583–9. 10.3892/ol.2011.300 PMC3406435 22848232

[147] Wennerberg E Kremer V Childs R Lundqvist A. CXCL10-Induced Migration of Adoptively Transferred Human Natural Killer Cells Toward Solid Tumors Causes Regression of Tumor Growth in Vivo. Cancer Immunol Immunother (2015) 64(2):225–35. 10.1007/s00262-014-1629-5 PMC11028951 25344904

[148] Somanchi SS Lee DA. Ex Vivo Expansion of Human NK Cells Using K562 Engineered to Express Membrane Bound IL21. Methods Mol Biol (2016) 1441:175–93. 10.1007/978-1-4939-3684-7_15 27177666

[149] Faden DL Concha-Benavente F Chakka AB McMichael EL Chandran U Ferris RL. Immunogenomic Correlates of Response to Cetuximab Monotherapy in Head and Neck Squamous Cell Carcinoma. Head Neck (2019) 41(8):2591–601. 10.1002/hed.25726 PMC6625877 30828910

[150] Socinski MA Jotte RM Cappuzzo F Orlandi F Stroyakovskiy D Nogami N. Atezolizumab for First-Line Treatment of Metastatic Nonsquamous NSCLC. New Engl J Med (2018) 378(24):2288–301. 10.1056/NEJMoa1716948 29863955

[151] Burtness B Harrington KJ Greil R Soulieres D Tahara M de Castro G Jr. Pembrolizumab Alone or With Chemotherapy *Versus* Cetuximab With Chemotherapy for Recurrent or Metastatic Squamous Cell Carcinoma of the Head and Neck (KEYNOTE-048): A Randomised, Open-Label, Phase 3 Study. Lancet (2019) 394(10212):1915–28. 10.1016/S0140-6736(19)32591-7 31679945

[152] Pesce S Greppi M Tabellini G Rampinelli F Parolini S Olive D. Identification of a Subset of Human Natural Killer Cells Expressing High Levels of Programmed Death 1: A Phenotypic and Functional Characterization. J Allergy Clin Immunol (2017) 139(1):335–346 e3. 10.1016/j.jaci.2016.04.025 27372564

[153] Riley JL. PD-1 Signaling in Primary T Cells. Immunological Rev (2009) 229(1):114–25. 10.1111/j.1600-065X.2009.00767.x PMC3424066 19426218

[154] Benson DM Jr Bakan CE Mishra A Hofmeister CC Efebera Y Becknell B. The PD-1/PD-L1 Axis Modulates the Natural Killer Cell *Versus* Multiple Myeloma Effect: A Therapeutic Target for CT-011, a Novel Monoclonal Anti-PD-1 Antibody. Blood (2010) 116(13):2286–94. 10.1182/blood-2010-02-271874 PMC3490105 20460501

[155] MacFarlane AWT Jillab M Plimack ER Hudes GR Uzzo RG Litwin S. PD-1 Expression on Peripheral Blood Cells Increases With Stage in Renal Cell Carcinoma Patients and is Rapidly Reduced After Surgical Tumor Resection. Cancer Immunol Res (2014) 2(4):320–31. 10.1158/2326-6066.CIR-13-0133 PMC4007343 24764579

[156] Liu Y Cheng Y Xu Y Wang Z Du X Li C. Increased Expression of Programmed Cell Death Protein 1 on NK Cells Inhibits NK-Cell-Mediated Anti-Tumor Function and Indicates Poor Prognosis in Digestive Cancers. Oncogene (2017) 36(44):6143–53. 10.1038/onc.2017.209 PMC5671935 28692048

[157] Concha-Benavente F Kansy B Moskovitz J Moy J Chandran U Ferris RL. PD-L1 Mediates Dysfunction in Activated PD-1(+) NK Cells in Head and Neck Cancer Patients. Cancer Immunol Res (2018) 6(12):1548–60. 10.1158/2326-6066.CIR-18-0062 PMC6512340 30282672

[158] Sacco AG Chen R Ghosh D Wong DJL Worden FP Adkins D. An Open Label, Nonrandomized, Multi-Arm, Phase II Trial Evaluating Pembrolizumab Combined With Cetuximab in Patients With Recurrent/Metastatic (R/M) Head and Neck Squamous Cell Carcinoma (HNSCC): Results of Cohort 1 Interim Analysis. J Clin Oncol (2019) 37(15_suppl):6033–3. 10.1200/JCO.2019.37.15_suppl.6033

[159] Bonomo P Desideri I Loi M Mangoni M Sottili M Marrazzo L. Anti PD-L1 Durvalumab Combined With Cetuximab and Radiotherapy in Locally Advanced Squamous Cell Carcinoma of the Head and Neck: A Phase I/II Study (DUCRO). Clin Trans Radiat Oncol (2018) 9:42–7. 10.1016/j.ctro.2018.01.005 PMC5862684 29594250

[160] Zandberg DP. Avelumab With or Without Cetuximab in Treating Patients With Advanced Skin Squamous Cell Cancer . Available at: https://ClinicalTrials.gov/show/NCT03944941.

[161] Harjunpää H Guillerey C. TIGIT as an Emerging Immune Checkpoint. Clin Exp Immunol (2020) 200(2):108–19. 10.1111/cei.13407 PMC7160651 31828774

[162] Chauvin J-M Zarour HM. TIGIT in Cancer Immunotherapy. J ImmunoTherapy Cancer (2020) 8(2):e000957. 10.1136/jitc-2020-000957 PMC7477968 32900861

[163] Sanchez-Correa B Valhondo I Hassouneh F Lopez-Sejas N Pera A Bergua JM. DNAM-1 and the TIGIT/PVRIG/TACTILE Axis: Novel Immune Checkpoints for Natural Killer Cell-Based Cancer Immunotherapy. Cancers (Basel) (2019) 11(6). 10.3390/cancers11060877 PMC6628015 31234588

[164] Wu L Mao L Liu J-F Chen L Yu G-T Yang L-L. Blockade of TIGIT/CD155 Signaling Reverses T-Cell Exhaustion and Enhances Antitumor Capability in Head and Neck Squamous Cell Carcinoma. Cancer Immunol Res (2019) 7(10):1700–13. 10.1158/2326-6066.CIR-18-0725 31387897

[165] Nguyen TL-A Cuende J Preillon J Garnero L Rabolli V Wald N. Abstract 3161: Preparation of Aclinical Trial With a-TIGIT Antagonist Antibody EOS884448, Which Demonstrates Potent Preclinical Activity and Safe Toxicology Profile. Cancer Res (2020) 80(16 Supplement):3161–1. 10.1158/1538-7445.am2020-3161

[166] Sloan KE Eustace BK Stewart JK Zehetmeier C Torella C Simeone M. CD155/PVR Plays a Key Role in Cell Motility During Tumor Cell Invasion and Migration. BMC Cancer (2004) 4:73. 10.1186/1471-2407-4-73 15471548 PMC524493

[167] Kučan Brlić P Lenac Roviš T Cinamon G Tsukerman P Mandelboim O Jonjić S. Targeting PVR (CD155) and its Receptors in Anti-Tumor Therapy. Cell Mol Immunol (2019) 16(1):40–52. 10.1038/s41423-018-0168-y 30275538 PMC6318332

[168] Wu L Parton A Lu L Adams M Schafer P Bartlett JB. Lenalidomide Enhances Antibody-Dependent Cellular Cytotoxicity of Solid Tumor Cells in Vitro: Influence of Host Immune and Tumor Markers. Cancer Immunol Immunother (2011) 60(1):61–73. 10.1007/s00262-010-0919-9 20848094 PMC11029172

[169] O’Donnell JS Madore J Li XY Smyth MJ Li XY Das I. Tumor Intrinsic and Extrinsic Immune Functions of CD155. Semin Cancer Biol (2019). 10.1016/j.semcancer.2019.11.013 31883911

[170] Li XY Das I Lepletier A Addala V Bald T Stannard K. CD155 Loss Enhances Tumor Suppression *via* Combined Host and Tumor-Intrinsic Mechanisms. J Clin Invest (2018) 128(6):2613–25. 10.1172/JCI98769 PMC5983325 29757192

[171] Björkström NK Riese P Heuts F Andersson S Fauriat C Ivarsson MA. Expression Patterns of NKG2A, KIR, and CD57 Define a Process of CD56dim NK-Cell Differentiation Uncoupled From NK-Cell Education. Blood (2010) 116(19):3853–64. 10.1182/blood-2010-04-281675 20696944

[172] Katou F Ohtani H Watanabe Y Nakayama T Yoshie O Hashimoto K. Differing Phenotypes Between Intraepithelial and Stromal Lymphocytes in Early-Stage Tongue Cancer. Cancer Res (2007) 67(23):11195–201. 10.1158/0008-5472.CAN-07-2637 18056444

[173] André P Denis C Soulas C Bourbon-Caillet C Lopez J Arnoux T. Anti-NKG2A Mab is a Checkpoint Inhibitor That Promotes Anti-Tumor Immunity by Unleashing Both T and NK Cells. Cell (2018) 175(7):1731–1743.e13. 10.1016/j.cell.2018.10.014 30503213 PMC6292840

[174] Levy EM Sycz G Arriaga JM Barrio MM von Euw EM Morales SB. Cetuximab-Mediated Cellular Cytotoxicity is Inhibited by HLA-E Membrane Expression in Colon Cancer Cells. Innate Immun (2009) 15(2):91–100. 10.1177/1753425908101404 19318419

[175] Segal NH Naidoo J Curigliano G Patel S Sahebjam S Papadopoulos KP. First-in-Human Dose Escalation of Monalizumab Plus Durvalumab, With Expansion in Patients With Metastatic Microsatellite-Stable Colorectal Cancer. J Clin Oncol (2018) 36(15_suppl):3540–0. 10.1200/JCO.2018.36.15_suppl.3540

[176] Rossin T. Monalizumab to Advance to Phase III in Head and Neck Cancer. France: ATCG Press Marseille (2019).

[177] Trivedi S Concha-Benavente F Srivastava RM Jie HB Gibson SP Schmitt NC. Immune Biomarkers of Anti-EGFR Monoclonal Antibody Therapy. Ann Oncol (2015) 26(1):40–7. 10.1093/annonc/mdu156 PMC4269339 24997207

[178] Vey N Karlin L Sadot-Lebouvier S Broussais F Berton-Rigaud D Rey J. A Phase 1 Study of Lirilumab (Antibody Against Killer Immunoglobulin-Like Receptor Antibody KIR2D; IPH2102) in Patients With Solid Tumors and Hematologic Malignancies. Oncotarget (2018) 9(25):17675–88. 10.18632/oncotarget.24832 PMC5915148 29707140

[179] Leidner R Kang H Haddad R Segal NH Wirth LJ Ferris RL. (2016)., in: Presented at: 2016 SITC Annual Meeting; November 9-13, 2016, National Harbor, MD, Abstract 456.

[180] Kohrt HE Thielens A Marabelle A Sagiv-Barfi I Sola C Chanuc F. Anti-KIR Antibody Enhancement of Anti-Lymphoma Activity of Natural Killer Cells as Monotherapy and in Combination With Anti-CD20 Antibodies. Blood (2014) 123(5):678–86. 10.1182/blood-2013-08-519199 PMC3907754 24326534

[181] Boudreau JE Hsu KC. Natural Killer Cell Education and the Response to Infection and Cancer Therapy: Stay Tuned. Trends Immunol (2018) 39(3):222–39. 10.1016/j.it.2017.12.001 PMC6013060 29397297

[182] Zhang J Mai S Chen H-M Kang K Li XC Chen S-H. Leukocyte Immunoglobulin-Like Receptors in Human Diseases: An Overview of Their Distribution, Function, and Potential Application for Immunotherapies. J Leukocyte Biol (2017) 102(2):351–60. 10.1189/jlb.5MR1216-534R PMC5505746 28351852

[183] Li NL Davidson CL Humar A Burshtyn DN. Modulation of the Inhibitory Receptor Leukocyte Ig-Like Receptor 1 on Human Natural Killer Cells. Front Immunol (2011) 2:46–6. 10.3389/fimmu.2011.00046 PMC3342057 22566836

[184] Roberti MP Julia EP Rocca YS Amat M Bravo AI Loza J. Overexpression of CD85j in TNBC Patients Inhibits Cetuximab-Mediated NK-Cell ADCC But can be Restored With CD85j Functional Blockade. Eur J Immunol (2015) 45(5):1560–9. 10.1002/eji.201445353 25726929

[185] Misumi T Tanabe K Fujikuni N Ohdan H. Stimulation of Natural Killer Cells With Rhcd137 Ligand Enhances Tumor-Targeting Antibody Efficacy in Gastric Cancer. PloS One (2018) 13(10):e0204880. 10.1371/journal.pone.0204880 30321186 PMC6188629

[186] Srivastava RM Trivedi S Concha-Benavente F Gibson SP Reeder C Ferrone S. CD137 Stimulation Enhances Cetuximab-Induced Natural Killer: Dendritic Cell Priming of Antitumor T-Cell Immunity in Patients With Head and Neck Cancer. Clin Cancer Res (2017) 23(3):707–16. 10.1158/1078-0432.CCR-16-0879 PMC5290200 27496866

[187] Chester C Sanmamed MF Wang J Melero I. Immunotherapy Targeting 4-1BB: Mechanistic Rationale, Clinical Results, and Future Strategies. Blood (2018) 131(1):49–57. 10.1182/blood-2017-06-741041 29118009

[188] Makkouk A Sundaram V Chester C Chang S Colevas AD Sunwoo JB. Characterizing CD137 Upregulation on NK Cells in Patients Receiving Monoclonal Antibody Therapy. Ann Oncol (2017) 28(2):415–20. 10.1093/annonc/mdw570 PMC6246233 27831501

[189] Qi X Li F Wu Y Cheng C Han P Wang J. Optimization of 4-1BB Antibody for Cancer Immunotherapy by Balancing Agonistic Strength With Fcγr Affinity. Nat Commun (2019) 10(1):2141–1. 10.1038/s41467-019-10088-1 PMC6526162 31105267

[190] Kohrt HE Houot R Weiskopf K Goldstein MJ Scheeren F Czerwinski D. Stimulation of Natural Killer Cells With a CD137-Specific Antibody Enhances Trastuzumab Efficacy in Xenotransplant Models of Breast Cancer. J Clin Invest (2012) 122(3):1066–75. 10.1172/JCI61226 PMC3287235 22326955

[191] Braunstein MJ Kucharczyk J Adams S. Targeting Toll-Like Receptors for Cancer Therapy. Target Oncol (2018) 13(5):583–98. 10.1007/s11523-018-0589-7 30229471

[192] Smits ELJM Ponsaerts P Berneman ZN Van Tendeloo VFI. The Use of TLR7 and TLR8 Ligands for the Enhancement of Cancer Immunotherapy. Oncologist (2008) 13(8):859–75. 10.1634/theoncologist.2008-0097 18701762

[193] Kaczanowska S Joseph AM Davila E. TLR Agonists: Our Best Frenemy in Cancer Immunotherapy. J leukocyte Biol (2013) 93(6):847–63. 10.1189/jlb.1012501 PMC3656332 23475577

[194] Stephenson RM Lim CM Matthews M Dietsch G Hershberg R Ferris RL. TLR8 Stimulation Enhances Cetuximab-Mediated Natural Killer Cell Lysis of Head and Neck Cancer Cells and Dendritic Cell Cross-Priming of EGFR-Specific CD8+ T Cells. Cancer Immunol Immunother (2013) 62(8):1347–57. 10.1007/s00262-013-1437-3 PMC3720845 23685782

[195] Lu H Dietsch GN Matthews M-AH Yang Y Ghanekar S Inokuma M. VTX-2337 is a Novel TLR8 Agonist That Activates NK Cells and Augments ADCC. Clin Cancer Res (2012) 18(2):499. 10.1158/1078-0432.CCR-11-1625 22128302

[196] Chow LQM Morishima C Eaton KD Baik CS Goulart BH Anderson LN. Phase Ib Trial of the Toll-Like Receptor 8 Agonist, Motolimod (VTX-2337), Combined With Cetuximab in Patients With Recurrent or Metastatic SCCHN. Clin Cancer Res (2017) 23(10):2442–50. 10.1158/1078-0432.CCR-16-1934 27810904

[197] Wu J Chen ZJ. Innate Immune Sensing and Signaling of Cytosolic Nucleic Acids. Annu Rev Immunol (2014) 32:461–88. 10.1146/annurev-immunol-032713-120156 24655297

[198] Abe T Barber GN. Cytosolic-DNA-Mediated, STING-Dependent Proinflammatory Gene Induction Necessitates Canonical NF-κb Activation Through TBK1. J Virol (2014) 88(10):5328–41. 10.1128/JVI.00037-14 PMC4019140 24600004

[199] Lohard S Bourgeois N Maillet L Gautier F Fétiveau A Lasla H. STING-Dependent Paracriny Shapes Apoptotic Priming of Breast Tumors in Response to Anti-Mitotic Treatment. Nat Commun (2020) 11(1):259. 10.1038/s41467-019-13689-y 31937780 PMC6959316

[200] Lemos H Mohamed E Huang L Ou R Pacholczyk G Arbab AS. STING Promotes the Growth of Tumors Characterized by Low Antigenicity *via* IDO Activation. Cancer Res (2016) 76(8):2076–81. 10.1158/0008-5472.CAN-15-1456 PMC4873329 26964621

[201] Chakraborty S Li L Puliyappadamba VT Guo G Hatanpaa KJ Mickey B. Constitutive and Ligand-Induced EGFR Signalling Triggers Distinct and Mutually Exclusive Downstream Signalling Networks. Nat Commun (2014) 5:5811. 10.1038/ncomms6811 25503978 PMC4268886

[202] Lu S Concha-Benavente F Shayan G Srivastava RM Gibson SP Wang L. STING Activation Enhances Cetuximab-Mediated NK Cell Activation and DC Maturation and Correlates With HPV(+) Status in Head and Neck Cancer. Oral Oncol (2018) 78:186–93. 10.1016/j.oraloncology.2018.01.019 PMC5877820 29496049

[203] Berraondo P Sanmamed MF Ochoa MC Etxeberria I Aznar MA Pérez-Gracia JL. Cytokines in Clinical Cancer Immunotherapy. Br J Cancer (2019) 120(1):6–15. 10.1038/s41416-018-0328-y 30413827 PMC6325155

[204] Kloss S Chambron N Gardlowski T Weil S Koch J Esser R. Cetuximab Reconstitutes Pro-Inflammatory Cytokine Secretions and Tumor-Infiltrating Capabilities of Smica-Inhibited NK Cells in HNSCC Tumor Spheroids. Front Immunol (2015) 6:543. 10.3389/fimmu.2015.00543 26579120 PMC4629470

[205] Morisaki T Umebayashi M Kiyota A Koya N Tanaka H Onishi H. Combining Cetuximab With Killer Lymphocytes Synergistically Inhibits Human Cholangiocarcinoma Cells in Vitro. Anticancer Res (2012) 32(6):2249–56. 22641659

[206] Hara M Nakanishi H Tsujimura K Matsui M Yatabe Y Manabe T. Interleukin-2 Potentiation of Cetuximab Antitumor Activity for Epidermal Growth Factor Receptor-Overexpressing Gastric Cancer Xenografts Through Antibody-Dependent Cellular Cytotoxicity. Cancer Sci (2008) 99(7):1471–8. 10.1111/j.1349-7006.2008.00821.x PMC11159884 18422755

[207] Zhao J Zhao J Perlman S. Differential Effects of IL-12 on Tregs and non-Treg T Cells: Roles of IFN-Γ, IL-2 and IL-2R. PloS One (2012) 7(9):e46241–1. 10.1371/journal.pone.0046241 PMC3459844 23029447

[208] Skrombolas D Frelinger JG. Challenges and Developing Solutions for Increasing the Benefits of IL-2 Treatment in Tumor Therapy. Expert Rev Clin Immunol (2014) 10(2):207–17. 10.1586/1744666X.2014.875856 PMC4072333 24410537

[209] Verma A Mathur R Farooque A Kaul V Gupta S Dwarakanath BS. T-Regulatory Cells in Tumor Progression and Therapy. Cancer Manag Res (2019) 11:10731–47. 10.2147/CMAR.S228887 PMC6935360 31920383

[210] Lee SC Lopez-Albaitero A Ferris RL. Immunotherapy of Head and Neck Cancer Using Tumor Antigen-Specific Monoclonal Antibodies. Curr Oncol Rep (2009) 11(2):156–62. 10.1007/s11912-009-0023-5 19216848

[211] Luedke E Jaime-Ramirez AC Bhave N Roda J Choudhary MM Kumar B. Cetuximab Therapy in Head and Neck Cancer: Immune Modulation With Interleukin-12 and Other Natural Killer Cell-Activating Cytokines. Surgery (2012) 152(3):431–40. 10.1016/j.surg.2012.05.035 PMC3432674 22770960

[212] Duggan MC Campbell AR McMichael EL Opheim KS Levine KM Bhave N. Co-Stimulation of the Fc Receptor and Interleukin-12 Receptor on Human Natural Killer Cells Leads to Increased Expression of Cd25. Oncoimmunology (2018) 7(2):e1381813. 10.1080/2162402X.2017.1381813 29308301 PMC5749624

[213] McMichael EL Benner B Atwal LS Courtney NB Mo X Davis ME. A Phase I/II Trial of Cetuximab in Combination With Interleukin-12 Administered to Patients With Unresectable Primary or Recurrent Head and Neck Squamous Cell Carcinoma. Clin Cancer Res (2019) 25(16):4955–65. 10.1158/1078-0432.CCR-18-2108 PMC6697573 31142501

[214] Feng T Cao AT Weaver CT Elson CO Cong Y. Interleukin-12 Converts Foxp3+ Regulatory T Cells to Interferon-Γ-Producing Foxp3+ T Cells That Inhibit Colitis. Gastroenterology (2011) 140(7):2031–43. 10.1053/j.gastro.2011.03.009 PMC3109200 21419767

[215] Choi SS Chhabra VS Nguyen QH Ank BJ Stiehm ER Roberts RL. Interleukin-15 Enhances Cytotoxicity, Receptor Expression, and Expansion of Neonatal Natural Killer Cells in Long-Term Culture. Clin Diagn Lab Immunol (2004) 11(5):879–88. 10.1128/CDLI.11.5.879-888.2004 PMC515280 15358647

[216] Rocca YS Roberti MP Julia EP Pampena MB Bruno L Rivero S. Phenotypic and Functional Dysregulated Blood NK Cells in Colorectal Cancer Patients can be Activated by Cetuximab Plus IL-2 or IL-15. Front Immunol (2016) 7:413. 10.3389/fimmu.2016.00413 27777574 PMC5056190

[217] Roberti MP Barrio MM Bravo AI Rocca YS Arriaga JM Bianchini M. IL-15 and IL-2 Increase Cetuximab-Mediated Cellular Cytotoxicity Against Triple Negative Breast Cancer Cell Lines Expressing EGFR. Breast Cancer Res Treat (2011) 130(2):465–75. 10.1007/s10549-011-1360-2 21308409

[218] Pinette A McMichael E Courtney NB Duggan M Benner BN Choueiry F. An IL-15-Based Superagonist ALT-803 Enhances the NK Cell Response to Cetuximab-Treated Squamous Cell Carcinoma of the Head and Neck. Cancer Immunol Immunother (2019) 68(8):1379–89. 10.1007/s00262-019-02372-2 PMC7032639 31338557

[219] Robinson TO Schluns KS. The Potential and Promise of IL-15 in Immuno-Oncogenic Therapies. Immunol Lett (2017) 190:159–68. 10.1016/j.imlet.2017.08.010 PMC5774016 28823521

[220] Romee R Cooley S Berrien-Elliott MM Westervelt P Verneris MR Wagner JE. First-in-Human Phase 1 Clinical Study of the IL-15 Superagonist Complex ALT-803 to Treat Relapse After Transplantation. Blood (2018) 131(23):2515–27. 10.1182/blood-2017-12-823757 PMC5992862 29463563

[221] Fehniger TA Hess BT Bachanova V Becker-Hapak M McClain E Berrien-Elliott M. Abstract CT146: First-in-Human Phase I Combination of the IL-15 Receptor Super Agonist Complex ALT-803 With a Therapeutic (Anti-CD20) Monoclonal Antibody (Mab) for Patients With Relapsed or Refractory Indolent non-Hodgkin Lymphoma (Inhl) . Cancer Res (2018) 78(13 Supplement):CT146–6. 10.1158/1538-7445.AM2018-CT146

[222] Moroz A Eppolito C Li Q Tao J Clegg CH Shrikant PA. IL-21 Enhances and Sustains CD8+ T Cell Responses to Achieve Durable Tumor Immunity: Comparative Evaluation of IL-2, IL-15, and IL-21. J Immunol (2004) 173(2):900–9. 10.4049/jimmunol.173.2.900 15240677

[223] McMichael EL Jaime-Ramirez AC Guenterberg KD Luedke E Atwal LS Campbell AR. IL-21 Enhances Natural Killer Cell Response to Cetuximab-Coated Pancreatic Tumor Cells. Clin Cancer Res (2017) 23(2):489–502. 10.1158/1078-0432.CCR-16-0004 27435400 PMC5241232

[224] Watanabe M Kono K Kawaguchi Y Mizukami Y Mimura K Maruyama T. Interleukin-21 can Efficiently Restore Impaired Antibody-Dependent Cell-Mediated Cytotoxicity in Patients With Oesophageal Squamous Cell Carcinoma. Br J Cancer (2010) 102(3):520–9. 10.1038/sj.bjc.6605502 PMC2822939 20029417

[225] Steele N Anthony A Saunders M Esmarck B Ehrnrooth E Kristjansen PEG. A Phase 1 Trial of Recombinant Human IL-21 in Combination With Cetuximab in Patients With Metastatic Colorectal Cancer. Br J Cancer (2012) 106(5):793–8. 10.1038/bjc.2011.599 PMC3305963 22315057

[226] Eskelund CW Nederby L Thysen AH Skovbo A Roug AS Hokland ME. Interleukin-21 and Rituximab Enhance NK Cell Functionality in Patients With B-Cell Chronic Lymphocytic Leukaemia. Leukemia Res (2011) 35(7):914–20. 10.1016/j.leukres.2011.02.006 21354618

[227] Vazquez-Lombardi R Roome B Christ D. Molecular Engineering of Therapeutic Cytokines. Antibodies (2013) 2(3):426–51. 10.3390/antib2030426

[228] Siddall E Khatri M Radhakrishnan J. Capillary Leak Syndrome: Etiologies, Pathophysiology, and Management. Kidney Int (2017) 92(1):37–46. 10.1016/j.kint.2016.11.029 28318633

[229] Sivakumar PV Garcia R Waggie KS Anderson-Haley M Nelson A Hughes SD. Comparison of Vascular Leak Syndrome in Mice Treated With IL21 or IL2. Comp Med (2013) 63(1):13–21. 23561933 PMC3567372

[230] Lord CJ Tutt AN Ashworth A. Synthetic Lethality and Cancer Therapy: Lessons Learned From the Development of PARP Inhibitors. Annu Rev Med (2015) 66:455–70. 10.1146/annurev-med-050913-022545 25341009

[231] Ding L Kim H-J Wang Q Kearns M Jiang T Ohlson CE. PARP Inhibition Elicits STING-Dependent Antitumor Immunity in Brca1-Deficient Ovarian Cancer. Cell Rep (2018) 25(11):2972–2980.e5. 10.1016/j.celrep.2018.11.054 30540933 PMC6366450

[232] Li T Chen ZJ. The Cgas-Cgamp-STING Pathway Connects DNA Damage to Inflammation, Senescence, and Cancer. J Exp Med (2018) 215(5):1287–99. 10.1084/jem.20180139 PMC5940270 29622565

[233] Boras I Nasser R Sabatinos S Antonescu CN. Signaling by the Epidermal Growth Factor Receptor Regulates DNA Repair. FASEB J (2019) 33(1_supplement):457.2. 10.1096/fasebj.2019.33.1_supplement.457.2.

[234] Nowsheen S Bonner JA Lobuglio AF Trummell H Whitley AC Dobelbower MC. Cetuximab Augments Cytotoxicity With Poly (Adp-Ribose) Polymerase Inhibition in Head and Neck Cancer. PloS One (2011) 6(8):e24148–8. 10.1371/journal.pone.0024148 PMC3166164 21912620

[235] Fenerty KE Padget M Wolfson B Gameiro SR Su Z Lee JH. Immunotherapy Utilizing the Combination of Natural Killer- and Antibody Dependent Cellular Cytotoxicity (ADCC)-Mediating Agents With Poly (ADP-Ribose) Polymerase (PARP) Inhibition. J Immunother Cancer (2018) 6(1):133. 10.1186/s40425-018-0445-4 30486888 PMC6264611

[236] Karam SD Reddy K Blatchford PJ Waxweiler T DeLouize AM Oweida A. Final Report of a Phase I Trial of Olaparib With Cetuximab and Radiation for Heavy Smoker Patients With Locally Advanced Head and Neck Cancer. Clin Cancer Res (2018) 24(20):4949–59. 10.1158/1078-0432.CCR-18-0467 PMC6873707 30084837

[237] Gillison ML Zhang Q Jordan R Xiao W Westra WH Trotti A. Tobacco Smoking and Increased Risk of Death and Progression for Patients With P16-Positive and P16-Negative Oropharyngeal Cancer. J Clin Oncol (2012) 30(17):2102–11. 10.1200/JCO.2011.38.4099 PMC3397696 22565003

[238] Ricks TK Chiu H-J Ison G Kim G McKee AE Kluetz P. Successes and Challenges of PARP Inhibitors in Cancer Therapy. Front Oncol (2015) 5:222–2. 10.3389/fonc.2015.00222 PMC4604313 26528434

[239] Kotla V Goel S Nischal S Heuck C Vivek K Das B. Mechanism of Action of Lenalidomide in Hematological Malignancies. J Hematol Oncol (2009) 2(1):36. 10.1186/1756-8722-2-36 19674465 PMC2736171

[240] Ghosh N Grunwald MR Fasan O Bhutani M. Expanding Role of Lenalidomide in Hematologic Malignancies. Cancer Manage Res (2015) 7:105–19. 10.2147/CMAR.S81310 PMC4427066 25999761

[241] Gamerith G Auer T Amann A Putzer D Schenk B Kircher B. Increase in Antibody-Dependent Cellular Cytotoxicity (ADCC) in a Patient With Advanced Colorectal Carcinoma Carrying a KRAS Mutation Under Lenalidomide Therapy. Cancer Biol Ther (2014) 15(3):266–70. 10.4161/cbt.27327 PMC3974827 24351336

[242] Krzewski K Bryceson YT. Molecular Mechanisms Regulating Cytotoxic Lymphocyte Development and Function, and Their Associations to Human Diseases. Front Immunol (2014) 5:279. 10.3389/fimmu.2014.00279 24966858 PMC4052198

[243] Bertino EM McMichael EL Mo X Trikha P Davis M Paul B. A Phase I Trial to Evaluate Antibody-Dependent Cellular Cytotoxicity of Cetuximab and Lenalidomide in Advanced Colorectal and Head and Neck Cancer. Mol Cancer Ther (2016) 15(9):2244–50. 10.1158/1535-7163.MCT-15-0879 PMC6805142 27458141

[244] Gandhi AK Shi T Li M Jungnelius U Romano A Tabernero J. Immunomodulatory Effects in a Phase II Study of Lenalidomide Combined With Cetuximab in Refractory KRAS-Mutant Metastatic Colorectal Cancer Patients. PloS One (2013) 8(11):e80437. 10.1371/journal.pone.0080437 24244687 PMC3823649

[245] ASCO Post. KEYNOTE-048: Pembrolizumab Monotherapy in Head and Neck Squamous Cell Carcinoma (2018). Available at: http://www.ascopost.com/News/59121.

[246] Yang J Li S Wang B Wu Y Chen Z Lv M. Potential Biomarkers for Anti-EGFR Therapy in Metastatic Colorectal Cancer. Tumour Biol (2016) 37(9):11645–55. 10.1007/s13277-016-5140-9 27422777

[247] Herbst RS Soria JC Kowanetz M Fine GD Hamid O Gordon MS. Predictive Correlates of Response to the Anti-PD-L1 Antibody MPDL3280A in Cancer Patients. Nature (2014) 515(7528):563–7. 10.1038/nature14011 PMC4836193 25428504

[248] Legrand FA Gandara DR Mariathasan S Powles T He X Zhang W. Association of High Tissue TMB and Atezolizumab Efficacy Across Multiple Tumor Types. J Clin Oncol (2018) 36(15_suppl):12000–0. 10.1200/JCO.2018.36.15_suppl.12000

[249] Donadon M Hudspeth K Cimino M Di Tommaso L Preti M Tentorio P. Increased Infiltration of Natural Killer and T Cells in Colorectal Liver Metastases Improves Patient Overall Survival. J Gastrointest Surg (2017) 21(8):1226–36. 10.1007/s11605-017-3446-6 28536806

[250] Balatoni T Mohos A Papp E Sebestyén T Liszkay G Oláh J. Tumor-Infiltrating Immune Cells as Potential Biomarkers Predicting Response to Treatment and Survival in Patients With Metastatic Melanoma Receiving Ipilimumab Therapy. Cancer Immunol Immunother (2018) 67(1):141–51. 10.1007/s00262-017-2072-1 PMC11028067 28988380

[251] Abdel-Rahman O Helbling D Schmidt J Petrausch U Giryes A Mehrabi A. Treatment-Related Death in Cancer Patients Treated With Immune Checkpoint Inhibitors: A Systematic Review and Meta-Analysis. Clin Oncol (R Coll Radiol) (2017) 29(4):218–30. 10.1016/j.clon.2016.11.007 27894673

[252] Simmet V Eberst L Marabelle A Cassier PA. Immune Checkpoint Inhibitor-Based Combinations: Is Dose Escalation Mandatory for Phase I Trials? Ann Oncol (2019) 30(11):1751–9. 10.1093/annonc/mdz286 31435659

